# Ultra‐Thin Flexible Encapsulating Materials for Soft Bio‐Integrated Electronics

**DOI:** 10.1002/advs.202202980

**Published:** 2022-08-28

**Authors:** Mingyu Sang, Kyubeen Kim, Jongwoon Shin, Ki Jun Yu

**Affiliations:** ^1^ School of Electrical and Electronic Engineering Yonsei University 50 Yonsei‐ro, Seodaemungu Seoul 03722 Republic of Korea; ^2^ YU‐KIST Institute Yonsei University 50 Yonsei‐ro, Seodaemungu Seoul 03722 Republic of Korea

**Keywords:** Thin film encapsulations, Flexible electronics, Wearable bioelectronics, Implantable bioelectronics, Biocompatible materials

## Abstract

Recently, bioelectronic devices extensively researched and developed through the convergence of flexible biocompatible materials and electronics design that enables  more precise diagnostics and therapeutics in human health care and opens up the potential to expand into various fields, such as clinical medicine and biomedical research. To establish an accurate and stable bidirectional bio‐interface, protection against the external environment and high mechanical deformation is essential for wearable bioelectronic devices. In the case of implantable bioelectronics, special encapsulation materials and optimized mechanical designs and configurations that provide electronic stability and functionality are required for accommodating various organ properties, lifespans, and functions in the biofluid environment. Here, this study introduces recent developments of ultra‐thin encapsulations with novel materials that can preserve or even improve the electrical performance of wearable and implantable bio‐integrated electronics by supporting safety and stability for protection from destruction and contamination as well as optimizing the use of bioelectronic systems in physiological environments. In addition, a summary of the materials, methods, and characteristics of the most widely used encapsulation technologies is introduced, thereby providing a strategic selection of appropriate choices of recently developed flexible bioelectronics.

## Graphical abstract

This review highlights the latest progress and trends, beginning with the development of advanced materials for encapsulations for flexible bioelectronics and the invention of new schemes for multifunctional flexible bio‐integrated electronics. By dividing this review into wearable and implantable electronics sections, it will help readers make appropriate choices and understand the use of materials and enable the development of various application fields.

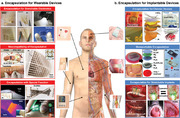

## 1 Introduction

Recent advances in flexible/stretchable bioelectronics research have opened up new directions of understanding the human body and delivering their new functionalities. These biomedical devices serve as not only for monitoring biological and physiological signals, but also for providing advancing treatment methodologies for incurable diseases.^[^ ^1^, ^2^, ^3^, ^4^, ^5^ ^]^ However, typical rigid healthcare devices show some limitations such as unreliable functions and tissue damages due to the imperfect device‐to‐tissue interface due to mechanical mismatch between device and tissue.^[^ ^6^, ^7^ ^]^ To overcome such limitations, flexible forms in bioelectronics have drawn attention and have been developed to adjust the electromechanical properties of the devices to match human skin or organ surfaces. Current flexible bioelectronics provide more stable and precise health monitoring and treatments by maintaining its mechanical softness in comparison with conventional rigid devices. Recently, various strategies, in structural design and composite materials, to form flexible bioelectronics have been intensively studied to acquire low mechanical modulus of the devices.^[^ ^8^, ^9^, ^10^, ^11^ ^]^ Low mechanical rigidity of the device establishes conformal contact to tissue interfaces, thereby not only preventing any irritation or tissue damage but also obtaining more accurate and precise signals from the tissue. Consequently, research in biomedical area has attempted to discover unidentified an origin of some diseases and suggest more specific or elaborate treatments. Moreover, the functionality of flexible bioelectronics continues to expand in a dynamical and specific manner over broad healthcare area.

To maintain flexibility, most bioelectronics are manufactured by thin film fabrication process forming substrate layer, functional electronics layer, and encapsulation layer. Each layer should be elaborately engineered in order to achieve desired electrical or mechanical properties according to purposes or working circumstances. Especially, the encapsulation layer of bioelectronics is of increasing importance as the range of applications widens, because failure of encapsulation causes device malfunction, current leakage, or even biological/mechanical tissue damage.^[^ ^12^, ^13^ ^]^ Therefore, all flexible bioelectronics are required to have suitable ultra‐thin encapsulations mainly for three objectives: device performance, device‐to‐tissue interface, and biomedical safety.

First, the encapsulation layer protects bioelectronics from external shocks and device damages due to surrounding environments (force, particles, oxidation, biofluids, etc.). Insufficient encapsulation can lead to a device performance degradation or an unexpected device failure.^[^ ^14^, ^15^ ^]^ To allow a bioelectronic device to obtain clear and reliable signals from tissues or organs or maintain actuation, the encapsulation layer should serve as a reliable barrier against the surrounding environment.^[^ ^16^ ^]^ Moreover, ultra‐thin encapsulations secure the device performance for flexible bioelectronics subject to external mechanical strain. Neutral mechanical plane, or neutral plane, is a conceptual layer that has zero strain under mechanical bending by neutralizing the forces of expansion and contraction.^[^ ^17^, ^18^, ^19^, ^20^ ^]^ Considering the mechanical properties of substrate and encapsulation materials, functional electronics can be placed on this neutral mechanical plane by adjusting the thicknesses of those two layers. As a result, the strain‐free electronics layer can maintain its functionality under large mechanical deformations.

Second, encapsulation that is surrounding the outermost part of the device is in direct contact with tissues by forming an adhesion or contact layer. As bioelectronics are supposed to operate through soft biological organic surface, the contact interfaces are critical in recording or stimulating circumstances. This emphasizes the importance of the encapsulation layer's specifications, such as thickness, roughness, or chemical composition, so that bioelectronics attached onto the tissues provide the stable functions. Ultrathin wearable electronics can be attached onto human skin via van der Waals force or electrostatic force, while implantable devices stick to wet surfaces of organs using hydrogen bonding, cation–*π* interaction, etc.^[^ ^21^, ^22^, ^23^ ^]^ This conformal and stable adhesion of the flexible bioelectronic devices onto highly deformable tissue is a key distinction from conventional rigid devices.

Third, when integrating conformal bioelectronics to human, biomedical safety should be considered carefully. The interfaces between the device and the tissue can often cause mechanical or biological problems such as skin irritation, abrasion, inflammation, or other immune responses.^[^ ^24^, ^25^ ^]^ This requires bioelectronics, especially implantable devices, to have a flexible/stretchable encapsulation layer composed of highly biocompatible materials. In addition, implantable devices should be proven biologically safe by biocompatibility test such as cytotoxicity, carcinogenicity, and immune response tests.^[^ ^26^ ^]^ Based on these considerations, the encapsulation layer requires sophisticated design that carefully considers functionality and the region of application.

**Figure 1:**
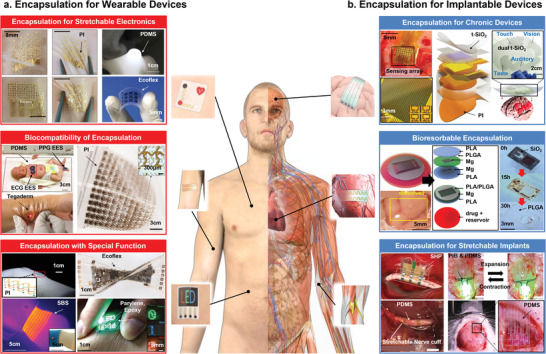
Encapsulation for wearable and implantable platform bioelectronics. Encapsulation for wearable bioelectronics enables them to be attached to human skin and perform important roles such as bio‐signal sensing normally and effectively. The appropriate selection and structural application of an encapsulation material protects the device from external environment and improves the device performance through various useful features such as flexibility, biocompatibility, transparency, and breathability. Implantable bioelectronics requires encapsulation for chronic operation in the body as well as bioresorbable encapsulation that dissolves in the body after operation for a certain period of time. In addition, the encapsulation layer should have sufficient flexibility to protect the device from biofluids, etc., and to allow the device to directly contact internal organs, tissues, and cells to monitor physiological information and successfully perform electrical stimulation. a) Encapsulation for stretchable: Four figures on the left hand side: Reproduced with permission.^[^ ^29^ ^]^ Copyright 2013, Springer Nature Limited. PDMS: Reproduced with permission.^[^ ^30^ ^]^ Copyright 2016, American Association for the Advancement of Science. Ecoflex: Reproduced with permission.^[^ ^31^ ^]^ Copyright 2018, Wiley‐VCH. Biocompatibility of encapsulation: Top left: Reproduced with permission.^[^ ^32^ ^]^ Copyright 2019, American Association for the Advancement of Science. Bottom left: Reproduced with permission.^[^ ^33^ ^]^ Copyright 2019, Wiley‐VCH. Right: Reproduced with permission.^[^ ^34^ ^]^ Copyright 2021, Springer Nature Limited. Encapsulation with special: Top left: Reproduced with permission.^[^ ^35^ ^]^ Copyright 2018, American Association for the Advancement of Science. Top right: Reproduced with permission.^[^ ^37^ ^]^ Copyright 2018, Springer Nature Limited. Bottom left: Reproduced with permission.^[^ ^36^ ^]^ Copyright 2015, American Chemical Society. Bottom right: Reproduced with permission.^[^ ^38^ ^]^ Copyright 2017, Wiley‐VCH. b) Encapsulation for chronic: Three figures on the left: Reproduced with permission.^[^ ^39^ ^]^ Copyright 2020, American Association for the Advancement of Science. Top and bottom right: Reproduced with permission.^[^ ^40^ ^]^ Copyright 2019, National Academy of Science. Bioresorbable encapsulation: Three figures on the left: Reproduced with permission.^[^ ^1^ ^]^ Copyright 2019, Springer Nature Limited. Three images on the right: Reproduced with permission.^[^ ^41^ ^]^ Copyright 2016, Springer Nature Limited. Encapsulation for strechable: Top left: Reproduced with permission.^[^ ^42^ ^]^ Copyright 2020, Springer Nature Limited. Top right: Reproduced with permission.^[^ ^43^ ^]^ Copyright 2019, Springer Nature Limited. Bottom left: Reproduced with permission.^[^ ^45^ ^]^ Copyright 2021, IOP Publishing. Bottom right: Reproduced with permission.^[^ ^44^ ^]^ Copyright 2021, Wiley‐VCH.

Flexible/stretchable bioelectronics can be divided into two major branches: wearable bioelectronics and implantable bioelectronics. Wearable bioelectronics, in engineering perspective, are electrical devices located onto/with a human skin or any wearable form of an equipment with a special role related to physiology, such as physiological signal recording, body‐movement recording, or delivering treatment. In this review, wearable bioelectronics in a flexible or stretchable form will be discussed, focusing on skin‐electronics. Another branch, implantable bioelectronics, are electrical devices, which are fully or partially implanted inside a human body by a surgical method, thereby providing various functions such as recording bio‐signals or actuating the organs or the tissues.^[^ ^27^, ^28^ ^]^ **Figure** **1** shows the recent advances in both flexible wearable and implantable bioelectronics focused on their encapsulation technology. Based on the purpose of protecting the device, the encapsulation layer is being developed to have specific properties. For some wearable electronics, the encapsulation layers are required to have stretchability and low moduli, because they should protect the electronic components from the outside environment and high mechanical deformation (wrinkle, bending, or stretching) of skin.^[^ ^29^, ^30^, ^31^ ^]^ The biocompatibility of encapsulation is essential for the bioelectronics, utilized as soft skin for premature infants to monitor their health precisely.^[^ ^32^, ^33^, ^34^ ^]^ In addition, for such a flexible photovoltaic electronic device, a transparent encapsulation is necessary when light source is applied. Some porous encapsulation layers are utilized for wearable platforms due to their superior skin ventilation or device‐cooling effect.^[^ ^35^, ^36^, ^37^, ^38^ ^]^ The encapsulation layers for implanted devices establish the lifetime under biofluidic environment. To acquire long‐term reliable physiological signals, advanced methods for construction of device systems with materials that show extremely low water or ion permeability demonstrate safe and chronic device operations.^[^ ^39^, ^40^ ^]^ Some implantable devices are made of composites which dissolve under biofluids environment, which obviates the procedure of the secondary surgery for device removal.^[^ ^1^, ^41^ ^]^ Nerves, blood vessels, and some organs continually expand and contract. Conformal bioelectronics implanted on these regions should have reasonably high mechanical stretchability while maintaining functionality under high deformations of the tissues.^[^ ^42^, ^43^, ^44^, ^45^ ^]^ Except satisfying those properties, encapsulation layers sometimes take a special role as one of bioelectronics components, such as cooling effect, self‐healing, insulator of capacitor, or drug delivery etc.^[^ ^46^, ^47^, ^48^, ^49^ ^]^

In this review, we summarize and discuss trending flexible/stretchable encapsulation materials, dividing the analysis by their properties. In Section 1, encapsulation materials for stretchability and other special properties are introduced for wearable platform electronics. In Section 2, encapsulation materials for implantable electronics platform are organized by the three major properties: chronical operation, biodegradability, and stretchability. After each section (1 and 2), encapsulation materials are summarized in terms of their key features and characteristics.

## 2 Encapsulation for Wearable Bioelectronics

Nowadays, the importance of wearable bioelectronics is increasing in various research fields, such as biomedical research, physiological research, disease prevention and treatment, clinical research, and human behavior research.^[^ ^22^, ^50^, ^51^, ^52^, ^53^, ^54^, ^55^, ^56^, ^57^, ^58^, ^59^, ^60^, ^61^, ^62^, ^63^, ^64^ ^]^ Recently, wearable devices that are capable of “sensing human bio‐signals” and “giving treatments to the body” are considered to play important roles.^[^ ^65^, ^66^, ^67^, ^68^, ^69^, ^70^, ^71^, ^72^, ^73^, ^74^, ^75^, ^76^, ^77^, ^78^, ^79^, ^80^, ^81^, ^82^, ^83^ ^]^ Functions of wearable bioelectronics include physical signal sensing such as strain, impedance, and pressure monitoring to detect human movement and body temperature monitoring, which are very important indicators for diseases and wounds;^[^ ^29^, ^68^, ^84^, ^85^, ^86^, ^87^, ^88^, ^89^, ^90^, ^91^, ^92^, ^93^, ^94^, ^95^, ^96^, ^97^, ^98^, ^99^, ^100^, ^101^, ^102^, ^103^ ^]^ electrophysiological sensing, such as electrocardiogram (ECG), electroencephalogram (EEG), and electrooculogram (EOG);^[^ ^33^, ^34^, ^46^, ^104^, ^105^, ^106^, ^107^, ^108^, ^109^, ^110^, ^111^, ^112^, ^113^, ^114^, ^115^, ^116^, ^117^, ^118^, ^119^ ^]^ and electrochemical sensing, which can detect body fluids such as sweat, tears, and saliva of the human body.^[^ ^120^, ^121^, ^122^, ^123^, ^124^, ^125^, ^126^, ^127^, ^128^, ^129^, ^130^, ^131^, ^132^, ^133^, ^134^, ^135^, ^136^, ^137^, ^138^, ^139^, ^140^, ^141^, ^142^, ^143^, ^144^, ^145^, ^146^, ^147^ ^]^ In addition, the functions and application targets of wearable devices are many such as wound care, acoustic devices such as ultrasound, actuators, simulators, and transparent devices many researchers are continuously conducting research to facilitate future technologies.^[^ ^30^, ^35^, ^36^, ^38^, ^148^, ^149^, ^150^, ^151^, ^152^, ^153^, ^154^, ^155^, ^156^, ^157^, ^158^, ^159^, ^160^, ^161^, ^162^, ^163^, ^164^, ^165^, ^166^, ^167^, ^168^, ^169^, ^170^, ^171^, ^172^, ^173^, ^174^, ^175^, ^176^, ^177^, ^178^ ^]^ Recently, in addition to medical applications, virtual reality/augmented reality (VR/AR) applications with wearable sensor technologies are being developed for body sensing and conformal haptic devices.^[^ ^179^, ^180^, ^181^, ^182^, ^183^ ^]^ To successfully perform such very important human target sensing and stimulation, accurate use and reliable operation of the wearable devices are essential when they are attached to human skin or various parts of the body. However, wearable devices often have difficulties in long‐term and stable operations due to exposure to external environmental factors such as heat, moisture, and wind, and other interference factors such as motion artifacts or hair. Therefore, the development of human‐friendly encapsulation technology for bioelectronics, which serves as a barrier that completely blocks ions, moisturefully adapts to the skin and body changes according to human movement, and sufficiently maintains the function and performance of the device, is very important while the device is conformally attaching to the skin.^[^ ^57^, ^184^ ^]^

**Figure 2:**
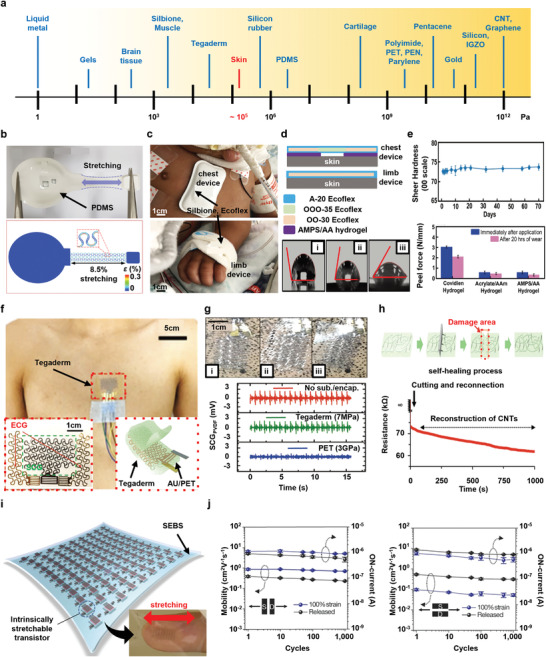
Stretchable encapsulation for wearable bioelectronics. a) Comparison of elastic moduli of various materials for wearable device fabrication and encapsulation layer construction and human skin. b) Image (top) and finite element analysis (FEA) simulation results (bottom) of stretching test of polydimethylsiloxane (PDMS)‐encapsulated device platform. Reproduced with permission.^[^ ^85^ ^]^ Copyright 2021, Springer Nature Limited. c) Picture of an interface device for a pediatric intensive care unit (PICU) patient softly encapsulated with Silbione and Ecoflex on the chest and limb. d) Packaging and encapsulation materials and layer structures for contact and compatibility with fragile and sensitive skin of PICU patients of chest and limb devices (top), and photos of water contact angle (bottom) to improve adhesion of encapsulation material: i) A‐20, ii) surfactant‐modified A‐20, and iii) AMPS/AA hydrogel. e) Mechanical stability test of dyed A‐20 with Shore hardness analysis (top) and peel force tests of three commercial hydrogel adhesives (bottom). Results for immediately after the sample was attached to the skin (blue) and after 20 h of wear (pink). Reproduced with permission.^[^ ^190^ ^]^ Copyright 2021, Wiley‐VCH. f) Tegaderm‐encapsulated ECG and SCG sensor platform attached to the chest and its structure illustration (inset). g) Comparative images and SCG signal measurement results according to substrate and encapsulation materials: i) no substrate/encapsulation, ii) Tegaderm, and iii) Polyethylene terephthalate (PET). Reproduced under the terms of a C‐CBC license.^[^ ^107^ ^]^ Copyright 2019, The Authors. Published by Wiley‐VCH. h) Self‐healing process and dynamic real‐time electrical reconstruction of carbon nanotubes (CNTs) encapsulated with the stretchable self‐healing polymer (SHP, PDMS–MPU_0.4_–IU_0.6_). Reproduced with permission.^[^ ^46^ ^]^ Copyright 2018, Springer Nature Limited. i) Styrene‐ethylene‐butylene‐styrene (SEBS) encapsulation and dielectric for stretchable functional wearable transistor arrays and an image of stretching the device on the finger (inset). j) Electrical properties and stretchability experiments of transistor array devices through stretching cycles of parallel (left) and perpendicular (right) deformations to the channel direction. Reproduced with permission.^[^ ^47^ ^]^ Copyright 2018, Springer Nature Limited.

**Figure 3:**
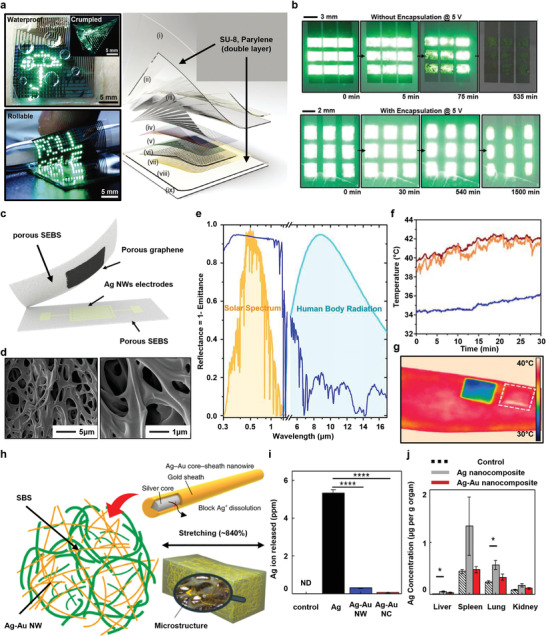
Encapsulation with special properties for wearable bioelectronics. a) Transparent encapsulation with SU‐8 and Parylene C for skin‐attached devices with integrated quantum dot display. b) Comparison of degradation of quantum dot light‐emitting diode (QLED) array without (top) and with (bottom) encapsulation. Reproduced with permission.^[^ ^38^ ^]^ Copyright 2017, Wiley‐VCH. c) Wearable electronic device using porous SEBS encapsulation with breathability. d) Scanning electron microscopy images of SEBS substrates with interconnected hierarchical pores. e) Spectral reflectance of nano/microporous SEBS encapsulation. f,g) Cooling effects by SEBS encapsulation as measured by thermal probe; skin (orange line), non‐porous SEBS‐attached skin (red line) and micro/nano‐porous SEBS‐attached skin (blue line) and infrared (IR) camera image. Reproduced with permission.^[^ ^48^ ^]^ Copyright 2019, National Academy of Science. h) Illustration of styrene‐butadiene‐styrene (SBS) encapsulated Ag–Au nanocomposite structure for improved stretchability and biocompatibility. i) Inductively coupled plasma mass spectrometry (ICP–MS) analysis results for Ag ions (Ag NW, Ag–Au NW and the Ag–Au nanocomposite incubated in Dulbecco's modified eagle medium). j) ICP‐MS analysis of Ag ions measured in various organs after the Ag nanocomposite and Ag–Au nanocomposite were implanted in the heart of a rat. Reproduced with permission.^[^ ^111^ ^]^ Copyright 2018, Springer Nature Limited.

Encapsulations for wearable devices can be classified according to their functions, as shown in **Figures** **2** and **3**. Figure 2 shows recent advances in technologies of stretchable encapsulations, which completely protect the wearable device from mechanical deformation of the skin and improve the performance of the device. Figure 2b introduces the encapsulation of a skin‐comfortable device using polydimethylsiloxane(PDMS); Figure 2c–e introduces various encapsulations, such as Silbione and Ecoflex, and research on their performance comparison; Figure 2f,g shows research on various encapsulations applied as tattoo‐like devices that are attached to the skin; and Figure 2h represents the encapsulation technology that reconstructs itself through self‐healing even if the device is damaged due to the movement of the human body or external shock, etc. Figure 2i,j shows the introduction, application, and performance of an encapsulation material that simultaneously functions as a dielectric as well as a stretchable encapsulation. Figure 3 represents various special functions of encapsulation for wearable devices. Figure 3a,b introduces a transparent, rollable, and waterproof encapsulation technology for wearable display devices; Figure 3c–f shows cooling and breathable encapsulation; and Figure 3h,i introduces a special encapsulation that provides biocompatibility and elasticity to non‐stretchable nanowires that are toxic to the human body.

### 2.1 Stretchable Encapsulation for Wearable Electronics

To minimize the effect of human body movement on wearable electronics, the device must be conformally attached to the skin and naturally accommodate mechanical deformation as it moves. The epidermis has an elastic modulus of several tens to hundreds of kPa and has an elastic strain of up to 15%.^[^ ^75^ ^]^ Therefore, to successfully operate a flexible wearable biosystem on the skin, it is essential to encapsulate the device using appropriate material with an elastic modulus similar to that of the target attachment site. Figure 2a presents the mechanical properties (e.g., Young's modulus) for various materials utilized as wearable platform as well as tissue and organs. However, the modulus difference, for exam between metal or silicon, and skin is extremely large. Therefore, the encapsulation of wearable devices using polymers with elastic properties that are similar to biological tissues and the development of optimized assembly and structure implementation methods could solve the problems caused by the mechanical mismatch between human tissue and the active layer of the device.

Figure 2b shows a skin‐attachable wearable sensor system for continuous long‐term monitoring of temperature and pressure at skin interfaces of various important parts of the body.^[^ ^85^ ^]^ This next‐generation biosensing system includes a thin encapsulation layer of PDMS, which protects internal electronic devices from mechanical deformation, biofluids, and electrical interference. Based on finite‐element analysis (FEA), in the case of stretching up to 8.5%, the strain applied to the copper layer of the device is much less than the yield strain (0.3%) due to the elastic polymer encapsulation. In addition to stretching, twisting ability of up to 270° and elastic (reversible) bending characteristics out of the plane up to a maximum bending radius of 7.3 mm ensure that the device can be gently attached to the skin without irritation, even in extreme human environments with a high risk of sacral ulceration but high curvature (elbow, heel, scapula, knee, sacrum, ankle, etc.). The electrical and mechanical design and implementation using biocompatible materials provides a suitable elastic response range, low stiffness, and sufficient rigidity for the device to fully attach to the skin and accommodate movement for monitoring of body temperature and pressure.

Non‐invasive wireless blood pressure monitoring helps in patient diagnosis and treatment by compensating for shortcomings such as ischemic risk and enabling natural body movement.^[^ ^186^, ^187^, ^188^, ^189^ ^]^ In pediatrics, since the skin is very sensitive and fragile, the development of a very safe, accurate, soft, and comfortable blood pressure monitoring method and device is essential. Figure 2c shows the continuous monitoring of vital signals by attaching an optimized wireless skin interface device, which can be used in the pediatric intensive care unit (PICU), to various parts of the infant's body.^[^ ^190^ ^]^ The wearable biosystem for special patients includes a stretchable encapsulation layer for safe and conformal contact with the skin interface of the child, as well as a barrier to prevent interference with the external environment and electrical noise. The device structure was designed with interface optimization for various conditions such as attachment site, size, and sensor type, as well as compatibility with fragile and sensitive skin. As shown in Figure 2d, the material for the inner filler layer is 00‐30 polyorganosiloxane elastomer (Ecoflex 00‐30, Smooth‐On), and the outer shell layer for encapsulation is A‐20 polyorganosiloxane elastomer (Silbione RTV 4420, Elkem). For the chest device, a soft hydrogel (KM 40A, Katecho) based on the 2‐acrylamido‐2‐methylpropane sulfonic acid/acrylic acid (AMPS/AA) copolymer, which serves as the conductive interface between the skin and the device, was used. Through FEA simulation, the rigidity and flexibility of the device system, including encapsulation for strain in multiple axes, bending at multiple angles, and twisting in various directions, were verified, and the effectiveness of structural design and material selection was also demonstrated. In addition, through the surface energy characteristics and the mechanical analysis of the interface, it was confirmed that the adhesive strength of the surfactant‐modified A‐20 and the compatibility with AMPS/AA hydrogel were improved. Safe and continuous use of the device in an intensive care unit environment requires sufficient stability and robustness of the encapsulation layer. Dyed A‐20 polyorganosiloxane elastomer exhibits long‐term mechanical stability under humidity and pressure conditions in Figure 2e. In addition, peel force test results of three types of hydrogel adhesives show that AMPS/AA hydrogel maintains the balance of interfacial softness and has advantages for soft skin contact during long‐term adhesion and continuous measurement. These simulation and experimental results suggest that, in terms of material, encapsulation stabilizes the performance of electronic devices in a specific environment such as a PICU environment, increases the compatibility of devices with sensitive pediatric skin and various attachment sites and sizes, and, ultimately, can improve patient diagnosis and treatment.

A polyvinylidene fluoride (Tegaderm)‐based skin‐soft electronic tattoos (e‐tattoos) sensor system for accurate and complete ECG and seismocardiography (SCG) mapping is shown in Figure 2f.^[^ ^107^ ^]^ An ultra‐thin sensor with a serpentine mesh structure can be attached to the chest to monitor blood pressure non‐invasively. For a device to measure biosignal changes continuously with a high signal‐to‐noise ratio in the chest, elasticity and flexibility must be achieved. Accordingly, Figure 2g shows a comparison experiment of substrate stiffness for three types of substrates and encapsulation layers to confirm these properties (i: sensor without substrate and encapsulation layer, ii: sensor using Tegaderm, and iii: sensor using polyethylene terephthalate (PET)). The 3D digital image correlation method was used to visualize the deformation of the skin surface, and the measurement result of the SCG signal for each type is shown. In addition, the effects of substrate and encapsulation on device sensitivity were further investigated through finite element modeling. Although the sensor with no substrate and encapsulation detects vibration more sensitively, it was found that it is appropriate to use Tegaderm as a substrate and encapsulation material to be applied as an e‐tattoo device and accept the deformation at the skin–material interface. Thanks to the flexible and stretchable ultra‐thin Tegaderm substrate and encapsulation layers, a tattoo‐like bioelectronic device system can conformally be attached to the skin of the chest, enabling accurate tracking of cardiac information.

Self‐healing electronics, which can be deformed and reconstructed with self‐healing chemistry have many advantages for wearable devices by mimicking the recovery properties of human skin; however, unlike self‐healable conductive polymers, conductive networks such as metal nanowire do not recover their elasticity and electrical conductivity after damage. Therefore, they cannot be used as self‐healing electrode modules. In Figure 2h, the conductive nanostructure network using a self‐healing polymer as an encapsulation layer after damage is reconstructed in order to recover conductivity and elasticity and exhibit high robustness and self‐healing power.^[^ ^46^ ^]^ The self‐healing encapsulation layer, carbon nanotube (CNT) network embedded with PDMS–4,4'‐methylenebis(phenylurea) unit (MPU)_0.4_–isophorone bisurea unit (IU)_0.6_, increases the resistance approximately tenfold after synthesis, but has a mechanical strain of over 50% and has autonomous self‐healing ability without external help after damage. In addition, AgNW/PDMS–MPU_0.4_–IU_0.6_ electrodes have high conductivity and flexibility and can be used as high‐performance wearable devices capable of self‐healing. The ultra‐thin wearable device using the nanocomposite material was conformally attached to the skin due to its low modulus, and it successfully and continuously performed light‐emitting diode (LED) driving, real‐time strain, and ECG monitoring. In addition, the device operated normally through self‐healing and reconfiguration despite repeated movement or damage. The stretchable self‐healing nanomaterial/polymer composite electrode with special encapsulation can be applied to wearable devices and used as unbreakable and very durable electronics in the future.

Encapsulation of wearable bio‐devices using various materials with high stretchability protects and preserves performance of the device under various conditions such as body fluids anddeformations, movements, and vibrations of the skin. It also provides comfort to wear, and due to the increase of the contact area, the stability and fidelity of the signal obtained from the skin is improved. However, in flexible electronic devices, the performance of the device may vary when the device is deformed under the mechanical stain. Therefore, recently, research has focused on maintaining device performance regardless of high deformation of the device. Figure 2i introduces a next‐generation skin electronic device using a polymer material with dielectric properties as well as its skin compatibility and high elasticity.^[^ ^47^ ^]^ That study shows an array of 347 transistors per square centimeter with high yield, excellent uniformity, and high device density. Styrene‐ethylene‐butylene‐styrene (SEBS), a polymer that retains dielectric and encapsulant properties without being damaged by large deformation, was used. As a result, it was possible to fabricate a large‐scale array of 6300 transistors in an area of ​​4.4 × 4.4 cm^2^ while maintaining high elasticity (up to 100%) and high charge carrier mobility (0.98 cm^2^ V^–1^ s^–1^). The fabricated stretchable wearable electronics device is translucent and can be conformally attached to the skin, as shown in the inset of Figure 2i. Figure 2j shows the results of 1000 cycles of 100% deformation in parallel and vertical to the channel direction of the elastic polymer‐based skin electronic device. The strain cycle test experiment revealed that the electronics with excellent stretchable encapsulation have stable charge carrier mobility with little current–voltage hysteresis. In addition, the device shows robustness and stable electrical performance in harsh environments such as mechanical stretching, pressure, torsion, and biaxial stretching. In that paper, Wang et al. introduced a polymer‐based transistor with average charge carrier mobility that is similar to that of amorphous silicon and demonstrate highly stretchable skin electronic devices by utilizing the active matrices. Polymers with elasticity, robustness, and electrical barrier properties have successfully performed the role of encapsulation layers and dielectrics in wearable devices, and the introduction of their manufacturing process methods has made it possible to apply them to electronic devices in the future.

### 2.2 Encapsulation with Special Properties for Wearable Bioelectronics

As technology advances and human health becomes more important, wearable bioelectronics with various functions are being continuously developed. In addition to monitoring bio‐signals, wearable devices with displays that show various vital signals or information, a power system‐based wearable electronics operated by sources from tissues and organs, and an acoustic device using piezoelectric properties have been actively studied and developed. Performing such unique functions requires proper engineering of the electronic element and the encapsulation layer, as well as design of an optimized structure for the wearable device. This section introduces encapsulation materials with unique properties and their applications.

Research visualizing various human bio‐information by skin‐attachable flexible  sensors system integrated with ultra‐thin quantum dot light‐emitting diode (QLED) displays and driving circuits are having a huge impact on next‐generation wearable electronic and optoelectronic devices. In this regard, proper encapsulation is essential for integration and application of QLED display, which has advantages such as high color purity, high electroluminescence brightness, and easy processability in wearable devices. In addition, to conformally attach to the user's skin to accommodate various deformations and reduce discomfort in movement, selection of a proper flexible and stable encapsulation material is necessary. As shown in Figure 3a, bilayer encapsulation of Parylene C/epoxy prevents mechanical damage and failure of QLED display in harsh mechanical environments such as extreme rolling, bending, and crumpling, and it minimizes the influence of the biofluid such as external moisture and sweat due to its excellent waterproof property.^[^ ^38^ ^]^ The utilization of multiple organic–inorganic encapsulation layers can overcome about long‐term stability concerns and ensure the operational stability of the device. As seen in Figure 3b, the robust encapsulation layer prevents degradation of the QLED display and enables long‐term stable operation when the device operates under an ambient condition of 5 V. It can also minimize the effect on the human skin due to the temperature rise of the high‐performance display. Visual transparency, which is the most important property of QLED display‐based wearable device encapsulation, enormously helps the integrated electronics to successfully represent bio‐information (body movement activity and temperature change) as various patterns, such as letters, numbers, and symbols, with satisfactory color and brightness.

The development of wearable devices is being carried out steadily, and potential materials for this purpose are also being explored. Among such research, securing the breathability of skin electronic that improve user comfort and minimize the risk of inflammation has been actively pursued in recent years. Figure 3c shows a wearable device based on a porous SEBS substrate and encapsulation layer fabricated through spray coating method.^[^ ^48^ ^]^ Figure 3d shows a scanning electron microscope (SEM) image of the fully interconnected hierarchical pores of the manufactured SEBS. Through a simple and low‐cost process, various pores with a size of ∼0.2–7 µm are formed and interconnected, resulting in very high elasticity and sunlight reflectance. Figure 3e presents the spectral reflectance of nano/micro‐sized porous SEBS. Special nanomaterials with stretchability and breathability create a synergistic effect of scattering the mid‐infrared rays of the human body and backscattering sunlight, which leads to excellent passive cooling. This activated, highly attractive material has passive cooling that provides a cooling effect of about 6 °C at the intensity of sunlight and is applied as a next‐generation wearable device that exhibits high breathability and waterproofness and even has recyclability. Figure 3f,g shows the cooling effect of porous SEBS encapsulation through thermal mapping using a thermal probe and infra‐red camera. The orange curve represents the temperature of skin exposed to sunlight, the red curve matches the temperature of the skin to which non‐porous SEBS was applied, and the blue curve shows the temperature of the skin to which the porous SEBS was applied. The wearable electronic device with an excellent cooling capable encapsulation layer can be utilized as a next‐generation human–machine interface that monitors biological signals such as temperature sensing, hydration sensing, pressure sensing, and electrophysiological sensing as well as acting as an electrical stimulator.

The most important characteristics of wearable devices in general are electrical conductivity, elasticity, and biocompatibility.^[^ ^39^, ^50^, ^51^, ^54^, ^55^, ^59^, ^61^, ^67^, ^69^, ^70^, ^71^, ^72^, ^76^, ^191^ ^]^ However, it is very difficult to find materials that satisfy all these requirements, synthesize them, and apply them to the bio system. Figure 3h shows that poly(styrene‐butadiene‐styrene) (SBS) elastomer synthesis and encapsulation are applied to highly conductive but toxic Ag–Au nanowires to impart elasticity and biocompatibility.^[^ ^111^ ^]^ The manufactured soft and flexible composite exhibits a nanostructure with 266% (maximum of 840%) elasticity based on phase separation, electrical conductivity of 41,850 S cm^−1^ (maximum of 72,600 S cm^−1^), and high biocompatibility and high oxidation resistance. A nano‐porous structure which acts as a cushion greatly increase softness and flexibility during stretching, lowering the Young's modulus while maintaining the electrical network stably. Figure 3i shows inductively coupled plasma mass spectrometry (ICP‐MS) analysis of Ag ion release from Ag nanowire, Ag–Au nanowire, and encapsulated Ag–Au nanowire composite incubated for 3 days in Dulbecco's modified eagle medium. Ag nanowires leached Ag ions at 5349 ppb, whereas Ag–Au nanowire composites showed only a trace level of 65 ppb Ag ions thanks to the external encapsulation and Au coating. In addition, as shown in Figure 3j, the results of implantation of Ag nanocomposite and Ag–Au nanocomposite in rat hearts for 3 weeks and measuring the distribution of Ag ions in other organs (liver, spleen, lung, and kidney) through ICP‐MS indicate that encapsulation effectively reduces the accumulation of Ag ions. Proper encapsulation is structurally compatible with conductive nanomaterials, giving them special properties such as biocompatibility, elasticity, mechanical softness, and oxidation resistance. In addition, wearable devices to with the encapsulation can be successfully applied to human, allowing continuous electrophysiological measurements, and can be developed as next‐generation bioelectronics that deliver electrical and thermal stimulation.

In summary, research and development of encapsulation technology for human compatibility and long‐term safety of wearable electronics with various functions (such as monitoring of various biological signals and human health, integration and compatibility with flexible displays, transmission of electrical and thermal stimuli, etc.) are very important. The encapsulation material and structural composition, including elasticity and flexibility, mechanical softness and firmness, biocompatibility, barrier properties against heat, moisture, ions, etc., adhesion, self‐healing ability, high transparency, and electrical properties help the wearable device reliably and accurately monitor the bio‐signals. **Table** **1** shows the comparison and arrangement of widely used encapsulation materials for wearable devices by the aforementioned important characteristics. Based on this, in the manufacture of wearable devices, proper selection of encapsulation materials and optimized structural design can improve device performance, which is one of the essential requirements for human health diagnosis and management, as well as human–machine interface development.

**Table 1:** Representative encapsulation materials for wearable bioelectronics and their properties

Materials	Encapsulation method	WVTR [g/m^2^⋅day]	OTR [cm^3^/m^2^⋅day⋅bar]	Elastic modulus [GPa]	Optical transmittance [%]	Glass transition temperature [°C]	Target application (Stretchability at device level)^a)^	Other properties	Refs.
Polydimethylsiloxane (PDMS)	Spin‐coating	65 (0.7 mm)	600–800 Barrer (0.7 mm)	(0.4–5) × 10^–3^	95.0 (1.5 µm)	125–150	Skin electronics, stretchable device (30–170%)	Thermal conductivity: 0.1 W (m⋅k)^−1^	[185, 192, 193, 194, 195]
Polyimide (PI)	Spin‐coating	0.4–21 (100 µm), 165 (25 µm)	301 (25 µm)	2.5	30–60 (100 µm), 90 (colorless PI)	450 (yellowish PI), 240–350 (colorless PI)	Skin electronics, flexible device (30% with serpentine structure)	Thermal conductivity: 0.18 W (m⋅k)^−1^	[98, 185, 196, 197, 198, 199]
Polyethylene terephthalate (PET)	Spin‐coating	7.9 (75 µm)	21 (75 µm)	2.3 ± 0.3	90.42 (100 µm)	77.3	Transparent electronics	Thermal conductivity: 0.19 W (m⋅k)^−1^	[184, 199, 200, 201]
Silbione	Spin‐coating	N/D	N/D	3×10^–6^ (50 µm)	N/D	N/D	Skin electronics, Stretchable device (60–168%)		[185, 203]
Ecoflex	Spin‐coating	135 (25 µm)	1200 (25 µm)	(20–125) × 10^–6^	82.0 (25 µm)	35–40	Skin electronics, Stretchable device (≈700%) (≈1780% after attaching the stretchable systems)		[185, 204, 205, 206]
Polyethylene naphthalate (PEN)	Spin‐coating	7.3 (100 µm)	3.0 (100 µm)	3.3 ± 0.4	87.02 (100 µm)	125	Transparent electronics		[185, 197, 201]
Styrene‐ethylene‐butylene‐styrene (SEBS)	Spin‐coating	2.9	79.3–96.2	0.8 × 10^–3^ (SEBS12), 6.2 × 10^–3^ (SEBS42), 17.65 ± 6.8 (SEBS)	≈82 (1 mm)	55	Skin electronics, stretchable device (≈100%)		[47, 192, 207, 208]
SU‐8	Spin‐coating	N/D	N/D	2.0 (SU‐8 2000)	>95	210	Transparent electronics, flexible device	Dielectrics constant: 3.28	[209, 210]
Parylene C	Spin‐coating	0.10	7.1	69 × 10^–3^	≈98 (1 µm)	13–80	Transparent electronics, flexible device	Dielectrics constant: 3.12, thermal conductivity: 0.084 W (m⋅k)^−1^	[211, 212]
Parylene F	Spin‐coating	0.32	34.7	55 × 10^–3^	Transparent	60–66	Transparent electronics, flexible device	Dielectrics constant: 2.20, thermal conductivity: 0.1 W (m⋅k)^−1^	[211]
Styrene‐butadiene‐styrene (SBS)	Spin‐coating	N/D	N/D	31.72 × 10^–3^	N/D	80	Skin electronics, stretchable device (≈2300%)	Thermoplastic elastomer	[213, 214]
Hydrogel	Casting	800–1100 (80Ch/20PEGF), 2250 (OHA/HA‐ADH)	2750–6000 (HA‐POL hydrogel)	13.28 ± 1.38 (80Ch/20PEGF)	≈96.05 (PVA hydrogel)	163 (PAMPS)	Skin electronics, stretchable device (≈4340%)		[193, 205, 215, 216, 217]

^a)^ Stretchability at device level is an example. It may be lower or higher depending on the structural design of the device

^b)^ WVTR: Water vapor transmission rate; OTR :Oxygen transmission rate.

## 3 Encapsulation for Implantable Bioelectronics

The implantable devices directly contact with organs, tissues, and cells in the body, which makes it possible to directly sense physiological information or stimulate specific tissues with high resolution,^[^ ^43^, ^217^, ^218^, ^219^, ^220^, ^221^, ^222^ ^]^ With this advantage, various physiological information including internal body pressure, strain on organs, and electrical signals from hearts or brains can be obtained and used for biomedical research.^[^ ^223^, ^224^, ^225^, ^226^, ^227^, ^228^, ^229^, ^230^ ^]^ In addition, disease treatment from stimulation of electricity or light, nerve regeneration or behavioral analysis can be undertaken.^[^ ^231^, ^232^, ^233^, ^234^ ^]^ Therefore, as a point of comparison with skin electronics, implantable bioelectronics can obtain much diverse and richer physiological information and even operate to treat diseases inside the body. However, as a degree of exposure to biofluids is very severe for implantable electronics, they require a much higher level of water and ion encapsulation barriers compared to those of skin electronics. Also, all materials consisting of implantable bioelectronics including encapsulation layers must be biocompatible to prevent damage to organs, tissues, and cells.^[^ ^231^, ^235^, ^236^, ^237^ ^]^ Depending upon the operating time, implantable devices with these advantages are divided into two types: long‐term implantable devices that operate semi‐permanently, and bioresorbable devices that dissolve and absorb into the body after operating certain period of time. Therefore, it is necessary to appropriately select the type, thickness, and deposition method of the encapsulation material according to the purpose of the researchers. In this part, each encapsulation material is introduced in detail by dividing it into two sections: long‐term and bioresorbable encapsulation.

### 3.1 Encapsulation for Long‐Term Implantable Bioelectronics

Encapsulation for chronic implantable bioelectronics requires long‐term stability in biofluid and intimate contact with soft and curvilinear biological tissue interfaces. Development of chronic bioencapsulation technology provides the potential for bioelectronics to function in the human body semi‐permanently. And, chronic bioencapsulation technology leads the bioelectronics to continuously sense biosignals and stimulate tissues or organs for diagnosis and treatment of diseases, which are important points in healthcare and biomedical fields. The chronic bioencapsulation layers are manufactured by integrating combinations of organic or inorganic materials with various deposition temperatures, thicknesses, and techniques such as spin‐coating, dip‐coating, chemical vapor deposition (CVD), or physical vapor deposition (PVD).^[^ ^16^, ^239^, ^240^, ^241^, ^242^, ^243^, ^244^ ^]^ Among the various materials, polymer layers (SU‐8, PDMS, Parylene C, etc.) can serve as long‐term bio‐encapsulations, formed comparatively simply and at low cost, but the layers need to be quite thick, ranging from tens to hundreds of microns because of the polymers’ high value of water vapor transmission rates (WVTRs).^[^ ^245^, ^246^, ^247^, ^248^, ^249^, ^250^ ^]^ Inorganic encapsulation layers (SiO_2_, SiN*_x_*, Al_2_O_3_, HfO_2_, etc.) deposited by atomic layer deposition (ALD) or CVD are not cost effective, but they show superior encapsulation properties compared to those of polymer, because inorganic materials have extremely low WVTRs.^[^ ^251^, ^252^, ^253^, ^254^ ^]^ However, even if the deposition quality of ALD or CVD is acceptable, perfect encapsulation on the entire surface of bioelectronics is almost impossible due to contamination or defects caused by limitations in the laboratory environment. Therefore, novel defect‐free materials and multi‐layer encapsulation have recently been studied to accomplish more perfect encapsulation.^[^ ^255^, ^256^, ^257^, ^258^, ^259^, ^260^ ^]^

**Figure 4:**
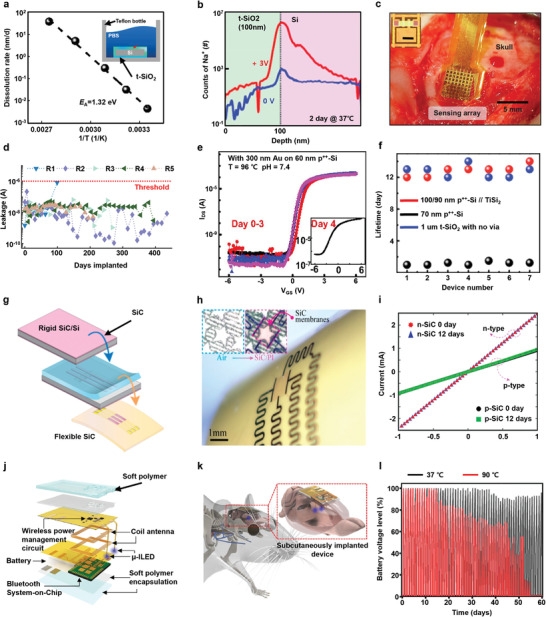
Encapsulation for long‐term implantable bioelectronics. a) Relation between t‐SiO_2_ dissolution rate and 1/T. (Inset) PBS soaking test of t‐SiO_2_. Reproduced with permission.^[^ ^261^ ^]^ Copyright 2016, National Academy of Science. b) Secondary‐ion‐mass‐spectroscopy (SIMS) result of sodium (Na^+^) penetration at the interface of t‐SiO_2_ and Si. Reproduced with permission.^[^ ^16^ ^]^ Copyright 2018, American Chemical Society. c) Image of 8 × 8 neural electrode array device subcutaneously implanted on brain of rat. d) Continuous measurement of leakage current of the implanted devices for 5 rats and the only implant in rat 1 showed failure. Reproduced with permission.^[^ ^39^ ^]^ Copyright 2020, American Association for the Advancement of Science. e) 96 °C PBS soak test of conductively coupled transistors with 300‐nm Au on 60‐nm p^++^‐Si. Inset shows failure of the device. Reproduced with permission.^[^ ^227^ ^]^ Copyright 2018, National Academy of Science. f) Lifetime comparison between encapsulated devices of p^++^‐Si//TiSi_2_ with via, p^++^‐Si with via, and t‐SiO_2_ with no via. Reproduced with permission.^[^ ^240^ ^]^ Copyright 2019, American Chemical Society. g) Schematic of SiC transferring process. h) Image of flexible SiC device wrapped on a glass rod. i) Stable current–voltage characteristics of p‐ and n‐type SiC films for 12 days of 96 °C PBS soaking. Reproduced with permission.^[^ ^240^ ^]^ Copyright 2019, American Chemical Society. j) Schematic of a polymer‐encapsulated fully implantable, soft, wirelessly rechargeable optoelectronic system. k) Schematic of a subdermally implanted wireless optoelectronic system on skull of freely‐moving mouse. l) Battery voltage levels of the device during 37 °C and 96 °C PBS soaking tests. Reproduced with permission.^[^ ^273^ ^]^ Copyright 2021, Springer Nature Limited.

Studies on defect‐free and uniform SiO_2_ thermally grown at extremely high temperature (1000–1100 °C) made it possible to resolve the issues of encapsulation layers deposited by ALD or CVD. The outstanding encapsulation quality of thermally grown SiO_2_ (t‐SiO_2_) was compared to different types of oxides formed by plasma enhanced chemical vapor deposition (PECVD) and electron beam evaporation.^[^ ^238^ ^]^ Inset of **Figure** **4**a represents Si wafer encapsulated by t‐SiO_2_ and soaked in phosphate buffered saline (PBS) solution. Accelerated tests were conducted by setting PBS solution for pH 7.4 and 70 °C. Dissolution kinetics of t‐SiO_2_ is SiO_2_ + 2H_2_O → Si(OH)_4_, which is induced by hydrolysis. The dissolution rates were determined to be exponentially depending on ≈1/T, and they were consistent with an activation energy of *E*_A_ = 1.32 eV and Arrhenius scaling. The results are shown in Figure 4a. From these results, simulation was performed, and dissolution rate of t‐SiO_2_ at pH 7.4 and 37 °C was found to be ∼4 × 10^–2^ nm d^−1^. This value means the lifetime of 1000‐nm‐thick t‐SiO_2_ layer is nearly 70 years, and this period is close to the lifespan of the patients who need chronic implantable bioelectronics.^[^ ^260^, ^261^ ^]^ As mentioned above, t‐SiO_2_ layer is an excellent water barrier, but a t‐SiO_2_ layer alone cannot achieve perfect encapsulation in biofluid because biofluid contains not only water, but also some species of ions such as potassium (K^+^) and sodium (Na^+^). As shown in Figure 4b, a 100‐nm‐thick sole t‐SiO_2_ layer does not effectively prevent ion diffusion after 2 days in PBS solution at 37 °C.^[^ ^16^ ^]^ Studies on multi‐layer encapsulation have been conducted to solve the issue, and additional deposition of Al_2_O_3_ or HfO_2_ by ALD, SiN*_x_* or Parylene C by CVD on t‐SiO_2_ successfully retarded ion diffusion and extended the lifetime of the bioencapsulation layers.^[^ ^16^, ^256^, ^262^ ^]^

Development of bioencapsulation based on t‐SiO_2_ made it possible for researchers to fabricate chronic implantable complementary metal oxide semiconductor (CMOS silicon nanomembrane (SiNM) transistors.^[^ ^20^, ^264^, ^265^ ^]^ Based on the fabricated SiNM transistors, active system devices with 18 columns and 22 rows of multiplex sensors (total 396) were fabricated, and the devices were chronically stable for at least 120 days in 37 °C PBS solution thanks to a 900‐nm‐thick t‐SiO_2_ encapsulation layer. The t‐SiO_2_ layer also served as interface for capacitive sensing between the electronics and adjacent biological tissues without presence of a direct metal pad. According to this study, ex‐vivo testing was conducted with Langendorff rabbit heart, and cardiac electrophysiology was successfully recorded through capacitive coupling of the transistors. The researchers conducted an in‐vivo study on rats and nonhuman primates to record high‐resolution brain electrophysiology data in a succeeding study, in which 8×8 neural electrode arrays encapsulated with 1 µm thick t‐SiO_2_ were implanted in five rodents to observe encapsulation lifetime and capacitive sensing performance as shown in Figure 4c.^[^ ^39^ ^]^ The result from the chronic experiment showed that four of five implanted devices remained stable under a 1 µA threshold voltage for a long time. Only one device failed operating in early days, which turned out to due to problems of data acquisition system. The lifetime of the devices ranged from 63 to 435 days, and the mean lifetime was 287 days, as shown in Figure 4d. These long‐lasting results from ex and in vivo studies guaranteed stability of t‐SiO_2_ encapsulation for capacitive coupling transistors.

Although capacitive coupling implantable devices showed long lifetimes, quality of electrophysiology was not sufficient. Therefore, the researchers developed another way for the improved quality of recording, which is the combination of highly doped SiNM (p^++^‐Si) with t‐SiO_2_ to serve both as bioencapsulation layer and direct conductively coupling interface through metal pad with brain tissues.^[^ ^227^ ^]^ For the test of lifetime of bioencapsulation layers, metal oxide semiconductor field effect transistor (MOSFET) encapsulated with 60‐nm p^++^‐Si and 1‐um t‐SiO_2_ with via was soaked in 96 °C PBS solution, and the MOSFET operated stably until 30 h. Similar devices with 300‐nm‐thick gold metal pad deposited on the 60‐nm p^++^‐Si side operated for 3 days in 96 °C PBS solution, as shown in Figure 4e, and this result indicates that electrode material can also serve as an encapsulation layer. Through Arrhenius scaling, lifetimes of 60‐nm p^++^‐Si without and with 300‐nm gold at 37 °C were 156 and 285 days, resepectively, and these lifetimes are sufficient for chronic neuroscience studies.

Several studies have demonstrated application p^++^‐Si as a long‐term bioencapsulation layer, but high dissolution rate of p^++^‐Si (0.47–5.0 nm d^−1^ in 37 °C, pH 7.4 PBS solution, 10^20^ cm^–3^ dopants) has a limitation in preventing the permanent operation of bioelectronics.^[^ ^266^ ^]^ Simply increasing the thickness of p^++^‐Si can extend the lifetime of the implantable devices, but replacing the p^++^‐Si to the material with a lower dissolution rate is a more effective approach. For this reason, a bilayer structure of titanium silicide (TiSi_2_) and p^++^‐Si is suggested. To fabricate the TiSi_2_ film, thin Ti film was deposited by electron beam evaporation on highly boron‐doped silicon‐on‐insulator (SOI). Then, the SOI was thermally annealed at 850 °C to derive a thin layer of TiSi_2_ and p^++^‐Si remaining underneath.^[^ ^240^ ^]^ The 100‐nm‐thick TiSi_2_ alloy has low sheet resistance, of ∼2.5 Ω sq^−1^, which means high conductivity. This high conductivity of TiSi_2_ enables direct conductive coupling of p^++^‐Si//TiSi_2_ with adjacent biotissues through a gold metal pad. NMOS transistors were encapsulated with p^++^‐Si//TiSi_2_ (100//90 nm) and via opened 1‐µm‐thick t‐SiO_2_. The transistors were soak tested in 96 °C PBS solution and functioned normally until 12 days. The failure of the device lies in complete dissolution of 1‐µm‐thick t‐SiO_2_ while TiSi_2_ remains stable, because dissolution rate of the 1‐µm‐thick t‐SiO_2_ is ≈90 nm d^−1^ and that of the 90‐nm‐thick TiSi_2_ is ≈3.3 nm d^−1^ in 96 °C PBS solution.^[^ ^261^ ^]^ In Figure 4f, lifetimes of three different types of the encapsulated devices were observed. Red dots are for via‐opened 1‐µm‐thick t‐SiO_2_ with p^++^‐Si//TiSi_2_ (100//90 nm), black dots are for via‐opened 1‐µm‐thick t‐SiO_2_ with 70 nm p^++^‐Si, and blue dots are for 1‐µm‐thick t‐SiO_2_ without via. The results show almost identical lifetime between two types of encapsulations (via‐opened t‐SiO_2_ with p^++^‐Si//TiSi_2_ and t‐SiO_2_ without via). This result supports the lifetime of the device mainly affected by the dissolution of t‐SiO_2_ because of extremely low dissolution rate of TiSi_2_.

Furthermore, single crystalline silicon carbide (SiC) is a novel material for the chronic implantable bioencapsulation due to its extremely low water and ion permeability and hydrolysis rate. Although SiC has excellent properties as encapsulation layer, its rigidity has, to date, blocked its application for flexible bioelectronics.^[^ ^267^, ^268^, ^269^ ^]^ In one study, the technique of physically transferring SiC nanomembranes on flexible bioelectronics for usage of outstanding bioencapsulation layer was developed;^[^ ^241^ ^]^ 230‐nm‐thick SiC nanomembranes were deposited on both sides of RCA‐cleaned Si wafers by using low‐pressure chemical vapor deposition (LPCVD) at a high temperature, of 1250 °C. Through conventional photolithography and dry and wet etching processes, free‐standing SiC NM was fabricated, and it was transferred to flexible polyimide (PI) substrate by physical process using PDMS stamp, as shown in Figure 4g. SiC NM transferred on flexible PI substrate was wrapped on a 12‐mm‐diameter glass rod, as shown in Figure 4h, and any negative effect was not observed with showing great flexibility. Helium leak test on SiC nanomembranes confirmed the absence of defects, and SiC‐encapsulated magnesium remained stable until 56 days in 96 °C PBS solution. Both p‐ and n‐type SiC nanomembranes showed stable electrical property until 12 days without influence of ion diffusion with a 0.14‐m concentration of Na^+^ in 96 °C PBS solution as shown in Figure 4i. From these results, SiC NM is among the longest living and perfect chronic bioencapsulation materials reported.

Polymer can be good alternative for oxide encapsulation layers thanks to its low‐cost and soft property.^[^ ^270^, ^271^, ^272^ ^]^ Polymer‐encapsulated fully implantable, soft, wirelessly rechargeable optoelectronic systems which are composed of neural probe, rechargeable battery, coil antenna, and Bluetooth chip have been suggested, as shown in Figure 4j.^[^ ^273^ ^]^ The fabricated device was implanted subdermally on skull of freely moving rats for in‐vivo studies, as shown in Figure 4k. The devices were encapsulated with special core/shell multiple polymer structure, and core layer was composed of bilayer of 600‐µm‐thick PDMS and 7‐µm‐thick Parylene C for biofluid encapsulation, and shell layer was 1400‐µm thick Ecoflex GEL for smooth interface. This core/shell structure provides three advantages: conformal contact between the device and curved skull surface, thermal dissipation of heat from the device operation to prevent adjacent tissue damage, and encapsulation of the device from biofluid. Due to these properties, the implanted devices could conformally contact onto rat skull with only 1.1 °C increase of body temperature, which is in the safe range. The devices were soak tested for 2 months at 90 °C in PBS, and they remain stable for 55 days as shown in Figure 4l. This indicates the lifetime of the device is about 1 year at 37 °C through Arrhenius scaling.

As mentioned above, long‐term implantable encapsulation layers developed from the state‐of‐the‐art materials and deposition techniques have been shown to be less influenced by water transmission, ion penetration, and hydrolysis in PBS. These results led to in vivo testing of bioimplantable devices even in nonhuman primates. Through the mentioned studies, lifetime of the implantable devices can be adjusted, ranging from several weeks to years, by determining the types and thicknesses of the encapsulation materials. These long‐lasting lifetimes guarantee to provide sufficient time to obtain information of medical science, neuroscience, cardiology, and electrophysiology.

### 3.2 Encapsulation for Bioresorbable Implantable Bioelectronics

Implanted bioelectronics should be taken out after a certain period of operation because of their side effects on the human body, such as irritations or infections. To prevent those problems, additional monitoring of implanted devices and secondary surgery for retrieval of implants are necessary.^[^ ^273^ ^]^ This puts the patient under associated burden and danger of infection from surgical re‐opening. Therefore, researchers are interested in the bioresorbable, or biodegradable, materials‐based bioelectronics.^[^ ^274^ ^]^ Encapsulation materials of bioresorbable electronics are basically composed of biodegradable natural polymer(polysaccharides), synthesized polymer (polyesters poly(lactic acid)(PLA), poly(glycolic acid)(PGA), and poly(lactic‐*co*‐glycolic acid)(PLGA)) or some of inorganic materials (SiO_2_).^[^ ^238^, ^275^, ^276^, ^277^ ^]^ Those materials are widely used as biodegradable substrates or encapsulation layers, protecting the implanted devices to maintain proper functionality. After performing their function for a designed period, the biodegradable substrate or encapsulation layer is meant to be dissolved in the body, resulting in device degradation.

**Figure 5:**
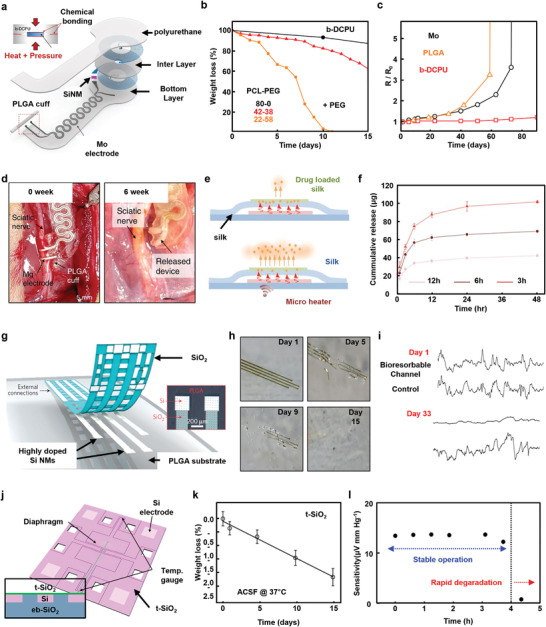
Encapsulation for bioresorbable implantable bioelectronics. a) Neural‐stimulating electrodes with poly(lactic‐co‐glycolic acid) (PLGA) and bioresorbable dynamic covalent polyurethane (b‐DCPU) encapsulations. b) Weight changes according to the immersion time in PBS (pH 7.4) at 37 °C with respect to PEG containing ratio, weight loss varies (polycaprolactone (PCL):polyethyleneglycol (PEG) = 80:0, black circle; PCL:PEG = 42:38, red triangle; PCL:PEG = 22‐58, orange rectangle). c) Normalized resistance changes of Mo electrodes according to the immersion time in PBS (pH 7.4) at 37 °C (without encapsulation, black circle; PLGA encapsulation, orange triangle; b‐DCPU encapsulation, red rectangle). d) Implanted image of bioresorbable PLGA nerve cuff electrodes. After 6 weeks, the nerve cuff is dissolved, leaving sciatic nerve. Reproduced with permission.^[^ ^280^ ^]^ Copyright 2020, Springer Nature Limited. e) Schematic of drug‐delivering device encapsulated with silk layer. Drug‐loaded silk on top of encapsulation layer releasing the drug with increasing temperature from micro heating system. f) Cumulative release amount according to time with regards to silk annealing time (3 h, 6 h, 12 h). Reproduced with permission.^[^ ^49^ ^]^ Copyright 2014, National Academy of Science. g) Bioresorbable electrodes array encapsulated with SiO_2_ encapsulation layer. h) Image of degradation in aqueous buffer solution (pH 10) at 37 °C. i) Recorded ECoG signal from bioresorbable electrode and controlled electrodes for 33 days. Reproduced with permission.^[^ ^290^ ^]^ Copyright 2016, Springer Nature Limited. j) Schematic of bioresorbable pressure sensor encapsulated with thermally grown silicon dioxide (t‐SiO_2_). k) Weight changes of t‐SiO_2_ layer according to the immersion time in ACSF at 37 °C. l) Sensitivity of implanted pressure sensor for about 4 h. Reproduced with permission.^[^ ^291^ ^]^ Copyright 2018, Springer Nature Limited.

Some synthetic polymers with biocompatibility and biodegradability are widely used for encapsulation layers of implanted bioelectronics.^[^ ^279^ ^]^ Compared to the natural polymers, they can be synthesized with controlled amount of composites, which brings the advantage of high reproducibility. PLGA is one of the widely used bioresorbable polymers group poly esters. **Figure** **5**a shows a bioresorbable electrical stimulator for a neural regeneration device with a PLGA cuff. To curve the electrical stimulation electrodes around the nerve, the device can be shaped into a cuff structure by hot‐pressing PLGA. However, because of its relatively short lifetime and swelling property under biofluidic environment, PLGA is not suitable for the therapeutic period of nerve regeneration.^[^ ^280^ ^]^ Therefore, other than poly‐ester group, specially synthesized polymer, so‐called bioresorbable dynamic covalent polyurethane (b‐DCPU), is used as an encapsulation and substrate layer of this device. This b‐DCPU is considered suitable for implanted bioelectronics because it has comparably high stretchability, comparably good adhesion property, and low permeability in biofluidic environment.^[^ ^281^, ^282^ ^]^ As with other synthetic polymers, the degrading ratio of this b‐DCPU is easily adjusted by its composition ratio Figure 5b. In this research, this b‐DCPU material is also used as a substrate layer. By applying pressure under heat, the two polymer layers can be easily bonded, providing better electrical performance of Mo electrodes. Figure 5c shows that, without encapsulation layer, the biodegradable electrode's resistance changes, while electrode encapsulated with b‐DCPU layer barely changes for 80 days. This result suggests that the electrodes functions as long‐term electrodes before being resorbed. Using this synthesized b‐DCPU, the implanted device enables proper operation over 30 days. Then, after the required periods, the PLGA cuff is resorbed and releases the sciatic nerve Figure 5d.

Natural polymers, such as cellulose, starch, chitosan, or silk, are known as useful in various biomedical applications because of their exceptional biocompatibility.^[^ ^283^, ^284^, ^285^ ^]^ Even though it is challenging to control their mechanical properties, there are some bioelectronics applications using natural polymers as encapsulation layer. Silk fibroin is one promising bioresorbable material with its adjustable lifetime.^[^ ^286^, ^287^ ^]^ By controlling the crystallinity of silk material, the lifetime can be expanded from minutes to several weeks. Figure 5e shows a diagram of silk‐encapsulated drug delivering electronics composed of fully bioresorbable materials.^[^ ^49^ ^]^ Mg and MgO are deposited on the silk substrate to form a bioresorbable electrical heater. In addition, another silk layer is formed as an encapsulation layer with controllable dissolution rate. The drug‐loaded silk layer is synthesized working as a drug delivery layer on top of the encapsulation layer. An external coil delivers electromagnetic power to the Mg coil inside the device, thereby heating up the device. With an increase in temperature, the drug‐loaded silk starts to dissolve inside the body rapidly and releases the drug. Figure 5f represents the cumulative release of drug stabilized in the crystallized silk matrix. According to the annealing time (3, 6, or 12 h), which affects the degree of crystallization of silk, the releasing ratio can be controlled. After the designated periods, the device is fully dissolved inside the body over 2 weeks. Beyond the bioresorbable encapsulation layer, natural polymers can be utilized for drug delivery medical applications.^[^ ^288^ ^]^

Although polymeric encapsulation is an attractive option for bioresorbable electronics application, their inherent hydrophilic nature results in swelling and water permeation.^[^ ^289^ ^]^ This leads to a preference of inorganic mateirlas for bioresorbable electronics as they offer slow dissolution rates without swelling effect under water environment, even though they have to be formed by chemical or physical deposition process.^[^ ^57^ ^]^ A thin layer of SiO_2_ formed by PECVD can serve as a bioresorbable dielectric or an encapsulation layer. Figure 5g shows an exploded diagram of a bioresorbable electrocorticography (ECoG) array, composed of PLGA substrate, highly doped SiNM electrodes, and SiO_2_ (PECVD) encapsulation layer.^[^ ^290^ ^]^ The SiO_2_ encapsulation layer as a bioresorbable bioelectronics encapsulation has a significant advantage over polymeric encapsulation, especially in a recording electrodes array. This inorganic layer functions as a barrier of biofluids between the interconnects and neural tissues, while it allows the recording sites to be attached on the neural tissue directly. In this regard, SiO_2_ layer is suitable for micropatterning by conventional photolithographic process with high resolution. Figure 5h shows the accelerated dissolution test. This bioresorbable ECoG array is immersed in PBS, pH 10, at 37 °C. Over two weeks, the device is fully dissolved, with SiO_2_ dissolution rate of 8.2 nm d^–1^. However, this dissolution time can be adjusted by controlling the thickness of the SiO_2_ encapsulation layer. For the in vivo experiment, they increased the thickness of SiO_2_ encapsulation (∼300 nm) for chronic brain signal recording. The recorded signal is compared to the control electrode, which is non‐biodegradable, and monitored the device functioning up to 32 days (Figure 5i).

Inorganics, such as SiO_2_, SiN_2_, or metal oxides, show preferable encapsulation performance, while blocking water permeation. However, the fabrication process of these inorganic encapsulation layer without any defect or crack is challenging, which has led to an alternative of t‐SiO_2_ encapsulation for bioresorbable electronics.^[^ ^239^, ^262^ ^]^ With their defects‐free property, adjustable thickness of t‐SiO_2_ layer shows high biofluidic blocking performance while being resorbed after enough working periods. Figure 5j shows an illustration of bioresorbable pressure sensor encapsulated by t‐SiO_2_ layer.^[^ ^291^ ^]^ With its high compatibility with conventional silicon fabrication process, the implantable pressure sensor uses micro‐scale air cavity structure to enhance the sensing performance. The thickness changes of this t‐SiO_2_ layer in artificial cerebrospinal fluid (ACSF) is about 120 times less than the changes of e‐beam deposited SiO_2_ layer (Figure 5k). This high endurance under biofluidic environment enables their use for long‐term implants, ensuring stable operation before dissolution. An ultrathin t‐SiO_2_ encapsulation layer was tested by monitoring the sensor sensitivity under 95 °C PBS solution. Figure 5l shows the results of stable device operation for 3 h, followed by rapid sensitivity deterioration. This shows that this defect‐free barrier can provide reliable protection, resulting in bare decline of device functionality. After designed periods, this bioelectronic pressure sensor fully dissolves in PBS solutions.

To alleviate patient burden, the role of bioresorbable medical devices will become more important. With proper material selection for encapsulation, the working period of the implants can be optimized and dissolved inside a body afterward. The material choice should be made after consideration of dissolution rate, mechanical property, and even the fabrication method, to be compatible with the underlying device's substrate layer and electrical components. Nowadays, regarding instant bioresorbable electronics, on‐demand bioresorbable property is being studied constantly.

### 3.3 Stretchable Encapsulation for Implantable Bioelectronics

Some human nerves, organs, and blood vessels work constantly, entailing a sort of periodical deformation. For this reason, there have been many difficulties in using biomedical devices in such areas.^[^ ^292^ ^]^ To handle this problem, some devices integrated hooks to be fixed on organs like bladder or heart. However, this method induces piercing damage on the organs, which can cause severe irritation. Surgical suturing is another option for fixation of bioelectronics. It can achieve minimum damage on the tissues, but a problem remains: Once bioelectronics are attached to tissues, they need to sustain their functionalities under high stretching stress from a target deformation. Low stretchability can lead to not just malfunction of the attached device, but also to contraction or squeezing force on the tissues causing irritation or even inflammation. Thus, the devices for implant on dynamic organs should have biocompatible and stretchable encapsulation layers.^[^ ^293^ ^]^

**Figure 6:**
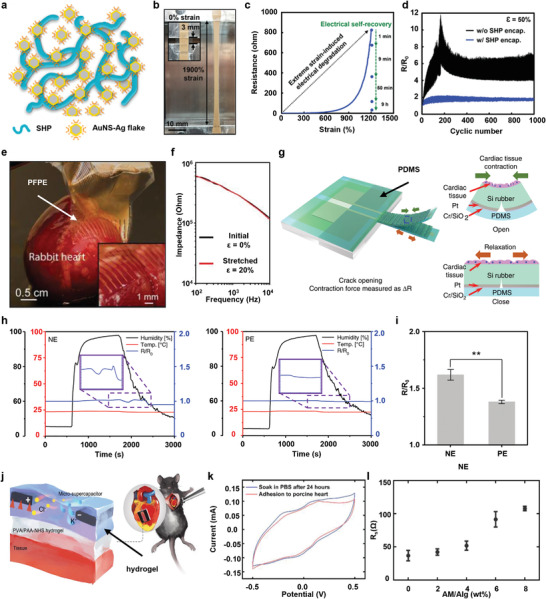
Encapsulation for stretchable implantable bioelectronics. a) Schematic of conductive nanomaterials (gold nanoshell and silver flakes) and self‐healing polymer composite. b) Image of 1900% stretching of nanocomposites encapsulated with self‐healing polymer. c) Resistance change of nanocomposite at up to 1200% stretching. The increased resistance is self‐recovered to its initial state after 9 h. d) Resistance change of nanocomposite over 1000 cycles (with encapsulation, blue; without encapsulation, black). Reproduced with permission.^[^ ^296^ ^]^ Copyright 2021, Wiley‐VCH. e) Image of stretchable array showing conformal attachment on the surface of rabbit heart. f) Impedance spectrum of a stretchable electrode under 20% stretching. Reproduced with permission.^[^ ^298^ ^]^ Copyright 2020, National Academy of Science. g) Schematics of stretchable crack sensor with PDMS encapsulation and its working principle. h) Performance of PDMS encapsulation layer by comparison of resistance stability under equal environment (humidity and temperature). i) Normalized resistance change with PDMS encapsulation (PE) and without encapsulation (NE). Reproduced with permission.^[^ ^300^ ^]^ Copyright 2020, Springer Nature Limited. j) Schematic of PVA/PAA‐NHS hydrogel encapsulated micro‐supercapacitor conformally attached on heart tissue. k) Cyclic voltammetry under PBS solution and on porcine heart. l) Measured resistances of different hydrogel composition (AM/Alg) ratios. Reproduced with permission.^[^ ^302^ ^]^ Copyright 2021, Wiley‐VCH.

Due to the periodic deformations of nerves, neuroprosthetics suffer from material degradation even if the device has some degree of stretchability. To overcome this problem, many studies have introduced self‐healing polymers as device encapsulation layers.^[^ ^293^, ^294^ ^]^ One example is a nanocomposite composed of gold nanoshell (AuNS) and silver flakes (Ag flake), which is mixed with a prepared self‐healing polymer, PDMS‐MPU_0.4_‐IU_0.6_ mixture (**Figure** **6**a).^[^ ^295^ ^]^ The composition ratio of AuNS–Ag flake and self‐healing polymer decides the mechanical and electrical properties to be optimized for the device application or the application region. Figure 6b shows this nanocomposite self‐healing polymer encapsulated device stretches extremely, up to ∼1900%. The resilience properties of this nanocomposite are evaluated by applying extreme mechanical deformation and relaxation while monitoring the electrical properties. Figure 6c shows that the fatigue‐resistant nanocomposite device can recover its original resistance in 9 h after ∼1200% stretching. Applying two self‐healing polymer encapsulation layers, the nanocomposite layers to substrate and encapsulation, this nanocomposite's electrical property is shown to be enhanced. Figure 6d represents an AuNS–Ag–SHP (gold nanoshell and silver flakes mixed with a self‐healing polymer) nanocomposite's cyclic stretching test with and without this self‐healing polymer encapsulation. The encapsulated one shows a highly stable response for the repetitive 50% strain cycles. With their high stretchable, resilient, and even self‐bonding properties, this self‐healing polymer‐based encapsulation layer is suitable for stretchable bioelectronics.

Heart is one of the most dynamic organs in human body, and the high mechanical stress induced on the attached electrodes is the major impediment on electrophysiological mapping. However, beyond the flexible electrode implantation, stretchable electrodes are emerging for this application based on investigations of micro‐patternable elastomers.^[^ ^297^ ^]^ Figure 6e shows an image of a stretchable electrode array for electrophysiological mapping attached to a porcine heart.^[^ ^298^ ^]^ To achieve high stretchability, they used a synthesized elastomer, diacrylate‐modified perfluoropolyether (PFPE‐DMA), as both substrate and encapsulation layer. This synthesized PFPE‐DMA can be micropatterned by conventional photolithography process, which enables the recording electrodes sites to be exposed and the interconnects to be passivated. The intrinsic stretchability and micro‐patternable property provide exceptional benefit by giving high spatial resolution compared to conventional stretchable electronics by structural design. In the electrochemical impedance measurement, the electrodes encapsulated by PFPE‐DMA have bare changes under 20% strain applied (Figure 6f).

Silicone elastomer, especially PDMS and Ecoflex, is one of the most widely used encapsulating materials in implantable bioelectronics because of its outstanding properties of biocompatibility, stretchability, and transparency.^[^ ^43^, ^299^ ^]^ Usually, those elastomers are considered as passive encapsulation layers. However, by adjusting their stretchability, they can be functionally utilized in bioelectronic devices. A crack sensor is nowadays a popular biosensing platform for monitoring contractile force, such as cardiac contractility.^[^ ^300^ ^]^ For this method, PDMS can be used as it not only protects the device, but also improves its sensing stability. Figure 6g is an illustration and brief outline of the working principle of PDMS‐encapsulated cardiac crack sensor. When the cardiac tissue contracts, cracks occur in silicone rubber and platinum electrodes, resulting in resistance changes according to the degree of cracking. This method conventionally interprets the cracks filled with biofluids, which adversely degenerate the electronic components in the devices. The plasma‐bonded PDMS encapsulation layer prevents any fluids penetration into the cracks to achieve stable operation of this cantilever structure. Especially, under implanted situation, humidity change is one major variable. Figure 6h shows the comparison of two crack sensors, with and without a PDMS encapsulation layer. Under steady temperature variance, the non‐encapsulated crack sensor shows shaky resistance changes, whereas the signal from the encapsulated sensor appears stable. Temperature dependency according to encapsulation layer is also measured. Figure 6i represents a resistance change from rapid temperature increase by about 12 °C. The crack sensor without encapsulation layer shows resistance change of ∼170%, while the PDMS encapsulated sensor shows ∼120%. This is because PDMS is one of the materials with low thermal conductivity.

In addition to the stretchable property, wet‐adhesive property is another major issue for implantable encapsulation interfaces of the bioelectronics on the organ tissue.^[^ ^191^, ^301^ ^]^ Despite possessing high stretchability, implanted electronics are prone to be unstable with lack of adhesion on wet tissue interfaces. For this reason, hydrogel is another recommended material for implantable bioelectronics because of their high adhesive property in wet tissue environment and extremely low modulus. Figure 6j demonstrates the suggestion that the use of double‐network hydrogel as an encapsulation and electrolyte of implantable supercapacitor.^[^ ^302^ ^]^ That study made an encapsulation layer by synthesizing poly(vinyl alcohol)/poly(acrylic acid) (PVA/PAA)‐based hydrogel and *N*‐hydroxysuccinimide (NHS) ester bond resulting in PAA‐NHS ester group, which behaves as a biological glue. This hydrogel‐based adhesive layer acts as interlayer between the device and tissue, providing mechanical conformality and electrophysiological safety by constraining any leakage current into or out of the adhesive. Attached on the wet porcine heart, this hydrogel supercapacitor shows minor difference in electrochemical performance compared to the PBS soaking environment (Figure 6k). The electrolyte of supercapacitor is another hydrogel material. It is composed of a polyacrylamide‐alginate (PAM‐Alg) structure, which can provide desirable electrical and mechanical properties. As the acrylamide ratio increases, the overall resistance decreases while mechanical stretchability increases (Figure 6l). This shows that the hydrogel‐based electrolyte or encapsulation is a superb option, as it can give adjustable mechanical and electrical performance, securing high stretchability and wet‐adhesive property.

Like flexible electronics substitutes, conventional rigid medical implants with their superior mechanical property, stretchable electronics‐based implants are being highlighted. Especially, the encapsulation layer of such stretchable implants forms a direct interfacial layer and gets highest strain under organ deformation. For the stable operation of such implantable bioelectronics, the material must be selected in consideration of the mechanical properties of the encapsulation layer material, as well as the possible process method.

In the case of an implantable device, it is hard to manipulate the device after implantation. Encapsulation with appropriate substances and methods is essential to enable the implanted device to operate in an in vivo environment. Basically, used encapsulation materials for implantable bioelectronics are summarized in **Table** **2** with their material properties. However, their chemical and mechanical properties, especially of polymers, can be tailored by changes in factors such as multiple compositions or annealing temperature during the fabrication process.

**Table 2:** Representative encapsulation materials for implantable bioelectronics and their properties

Materials	Type	Encapsulation method	Dissolution rate	Target organs	Elastic modulus [GPa]	Refs.
Poly(lactic‐co‐glycolic acid) (PLGA)	Synthetic polymer	Casting	100% weight loss in 50 days (50/50 PLGA) at 37 °C, pH 7.4	Nerve cuff, heart	0.092	[232, 280, 303, 304, 305]
Polyurethane (PU)	Synthetic polymer	Casting	>80% weight loss in 4 days at 37 °C, pH 7.4	Nerve stimulator	≈0.037	[279, 305, 306]
PDMS	Synthetic polymer	Casting, spin coating	N/D	Brain, bladder spine	(1–2) × 10^–3^	[43, 273, 308, 309]
Ecoflex	Synthetic polymer	Casting, spin coating	N/D	Brain, eye	<0.1 × 10^–3^	[273, 310, 311]
Silk	Natural polymer	Casting	30% weight loss in 14 days at 37 °C, pH 7.4	Subcutaneous tissue	1	[49, 312, 313, 314]
t‐SiO_2_	Inorganic	Dry oxidation of Si	≈0.04 nm/d at 37 °C, pH 7.4	Brain, heart	60–100	[16, 227, 244, 261, 262, 264, 291, 315, 316, 317]
SiO_2_	Inorganic	PECVD	0.1–13.6 nm d^−1^ at 37 °C, pH 7.4	Brain, heart	60–100	[41, 239, 290, 291, 315, 316, 317, 318]
SiO_2_	Inorganic	ALD	≈0.08 nm d^−1^ at 37 °C, pH 7.4	Brain, heart	60–100	[239, 256, 315, 316, 317]
Si_3_N_4_	Inorganic	LPCVD	≈0.3 nm d^−1^ at 37 °C, pH 7.4	Brain, heart	140–290	[239, 319, 320, 321, 322]
p^++^‐Si	Inorganic	Doping of Si	0.47–0.50 nm d^−1^ at 37 °C, pH 7.4, 10^20^ cm^–3^ dopants	Brain, heart	4–180	[227, 244, 266, 323, 324]
TiSi_2_	Inorganic	Thermal annealing of Ti on Si	≈3.3 nm d^−1^ at 96 °C, pH 7.4	Brain, heart	250–280	[240, 325]
HfO_2_	Inorganic	ALD	≈8 um d^−1^ at 96 °C, pH 7.4	Brain, heart	150–260	[16, 256, 257, 308, 326, 327]

## 4 Challenges and Perspectives

Despite various advanced strategies for bioelectronic encapsulation, limitations remain to be addressed. Long‐term stability is one of the most important factors for flexible bioelectronics to expand their practical use as a chronic device. As a chronic encapsulation material, some inorganics were introduced with various deposition process methods. However, inorganic materials have inherently high rigidity and involve more demanding deposition processes than organic materials. Organics have relatively low rigidity and involve easy coating methods, but they show low barrier properties. To elongate the lifetime of electronics, multi‐layered of inorganic/inorganic or inorganic/organic encapsulation methods have been introduced, but they are still confined to ensuring limited‐time operation and a proof‐of‐concept level of encapsulation performance.^[^ ^247^, ^251^, ^327^ ^]^

Through the advances in material science and innovative manufacturing processes, it seems possible to create an ultra‐long‐term, or even a semi‐permanent, encapsulation technique. Moreover, as introduced in this review, the possibility of further utilizing the outermost layer of the flexible electronics by imparting a special functionality to an encapsulation layer is expected. From the electronics perspective, additional active operation of the outermost layer offers many opportunities of device utilization. Along with all perspectives of encapsulation layer, some ethical issues should be carefully discussed because human subject should be considered priority before the applied medical device even with a perfect encapsulation layer.^[^ ^329^, ^330^ ^]^

## 5 Conclusion

This article reviewed the most recent and novel encapsulation materials and methodologies applied in flexible bio‐integrated electronics. As introduced in this review, it is essential to select encapsulation materials appropriate to the purposes of two types of biomedical devices, wearable and implantable electronics. The section on the encapsulation of wearable electronics was divided into two parts, “stretchable encapsulation” and “encapsulation with special properties,” according to their unique characteristics. Especially, various polymers were highlighted for stretchable encapsulation materials focusing on their high intrinsic flexibility and stretchability, which can protect the electronics from large strain and deformation during dynamic movements of live animal models. In addition to flexibility and stretchability, special properties such as transparency, breathability, self‐healing capability, and low water permeability were introduced in the encapsulation with special properties section with various materials and applications. For the encapsulation of implantable electronics section, materials for long‐term and bioresorbable encapsulation were widely introduced. For long‐term implantable electronics, multi‐layers of thick polymers or organic materials are applied as encapsulation materials to effectively prevent biofluid from adhering to the device. In contrast to long‐term implantable encapsulation, materials for bioresorbable encapsulation dissolve and adsorb to bodies in biofluid after certain periods of operational time. These various encapsulation materials are summarized in the above tables with their special characteristics. This information will help future researchers choose their device structure, size, and target organs according to the proposed functions. The integration and development of these encapsulation materials will lead to noteworthy growth in the fields of materials science and biomedical and bioelectronic engineering.

## Conflict of Interest

The authors declare no conflict of interest.

## References

[1] J. Lee , H. R. Cho , G. D. Cha , H. Seo , S. Lee , C. K. Park , J. W. Kim , S. Qiao , L. Wang , D. Kang , T. Kang , T. Ichikawa , J. Kim , H. Lee , W. Lee , S. Kim , S. T. Lee , N. Lu , T. Hyeon , S. H. Choi , D. H. Kim , Nat. Commun. 2019, 10, 5205. 31729383 10.1038/s41467-019-13198-y PMC6858362

[2] F. P. Pons‐Faudoa , A. Ballerini , J. Sakamoto , A. Grattoni , Biomed. Microdevices 2019, 21, 47. 31104136 10.1007/s10544-019-0389-6 PMC7161312

[3] Y. Yang , M. Wu , A. Vazquez‐Guardado , A. J. Wegener , J. G. Grajales‐Reyes , Y. Deng , T. Wang , R. Avila , J. A. Moreno , S. Minkowicz , V. Dumrongprechachan , J. Lee , S. Zhang , A. A. Legaria , Y. Ma , S. Mehta , D. Franklin , L. Hartman , W. Bai , M. Han , H. Zhao , W. Lu , Y. Yu , X. Sheng , A. Banks , X. Yu , Z. R. Donaldson , R. W. t. Gereau , C. H. Good , Z. Xie , et al., Nat. Neurosci. 2021, 24, 1035. 33972800 10.1038/s41593-021-00849-x PMC8694284

[4] W. Wei , Y. Song , L. Wang , S. Zhang , J. Luo , S. Xu , X. Cai , Microsyst. Nanoeng. 2015, 1, 150002.

[5] Y. J. Hong , H. Jeong , K. W. Cho , N. Lu , D. H. Kim , Adv. Funct. Mater. 2019, 29, 1808247.

[6] G. Balakrishnan , J. Song , C. Mou , C. J. Bettinger , Adv. Mater. 2022, 34, 2106787. 10.1002/adma.202106787 PMC8917047 34751987

[7] C. Moffatt , S. Murray , V. Keeley , A. Aubeeluck , Int. Wound J. 2017, 14, 1305. 28857457 10.1111/iwj.12804 PMC7950059

[8] W. Wang , S. Yang , K. Ding , L. Jiao , J. Yan , W. Zhao , Y. Ma , T. Wang , B. Cheng , Y. Ni , Chem. Eng. J. 2021, 425, 129949.

[9] H. Kim , Y. S. Kim , M. Mahmood , S. Kwon , N. Zavanelli , H. S. Kim , Y. S. Rim , F. Epps , W. H. Yeo , Adv. Sci. 2020, 7, 2000810. 10.1002/advs.202000810 PMC7404159 32775164

[10] Z. Rao , Y. Lu , Z. Li , K. Sim , Z. Ma , J. Xiao , C. Yu , Nat. Electron. 2021, 4, 513.

[11] M. Sang , J. Shin , K. Kim , K. J. Yu , Nanomaterials 2019, 9, 374. 10.3390/nano9030374 PMC6474003 30841599

[12] L. Guo , Neural Interface Engineering, Springer, Cham 2020.

[13] P. L.e Floch , N. Molinari , K. Nan , S. Zhang , B. Kozinsky , Z. Suo , J. Liu , Nano Lett. 2020, 20, 224. 31775509 10.1021/acs.nanolett.9b03705

[14] T. S. Kim , H. J. Kim , J.‐H. Han , W. J. Choi , K. J. Yu , ACS Appl. Energy Mater. 2021, 5, 227.

[15] T. S. Kim , H. J. Kim , D. M. Geum , J. H. Han , I. S. Kim , N. Hong , G. H. Ryu , J. Kang , W. J. Choi , K. J. Yu , ACS Appl. Mater. Interfaces 2021, 13, 13248. 33691400 10.1021/acsami.1c00006

[16] E. Song , R. Li , X. Jin , H. Du , Y. Huang , J. Zhang , Y. Xia , H. Fang , Y. K. Lee , K. J. Yu , J. K. Chang , Y. Mei , M. A. Alam , Y. Huang , J. A. Rogers , ACS Nano 2018, 12, 10317. 30281278 10.1021/acsnano.8b05552

[17] H. E. Lee , S. Kim , J. Ko , H.‐I. Yeom , C.‐W. Byun , S. H. Lee , D. J. Joe , T.‐H. Im , S.‐H. K. Park , K. J. Lee , Adv. Funct. Mater. 2016, 26, 6170.

[18] D. Khodagholy , T. Doublet , M. Gurfinkel , P. Quilichini , E. Ismailova , P. Leleux , T. Herve , S. Sanaur , C. Bernard , G. G. Malliaras , Adv. Mater. 2011, 23, H268. 21826747 10.1002/adma.201102378

[19] T. Kinkeldei , K. Cherenack , C. Zysset , N. C. Woo , G. Tröster , Eur. Phys. J. Appl. Phys. 2011, 55, 23901.

[20] J. Viventi , D.‐H. Kim , J. D. Moss , Y.‐S. Kim , J. A. Blanco , N. Annetta , A. Hicks , J. Xiao , Y. Huang , D. J. Callans , J. A. Rogers , B. Litt , Sci. Transl. Med. 2010, 2, 24ra22. 10.1126/scitranslmed.3000738 PMC3039774 20375008

[21] X. Shi , P. Wu , Small 2021, 17, e2101220. 34105250 10.1002/smll.202101220

[22] J. W. Jeong , W. H. Yeo , A. Akhtar , J. J. Norton , Y. J. Kwack , S. Li , S. Y. Jung , Y. Su , W. Lee , J. Xia , H. Cheng , Y. Huang , W. S. Choi , T. Bretl , J. A. Rogers , Adv. Mater. 2013, 25, 6839. 24327417 10.1002/adma.201301921

[23] M. Suneetha , O. Sun Moo , S. Mo Choi , S. Zo , K. Madhusudana Rao , S. Soo Han , Chem. Eng. J. 2021, 426, 130847.

[24] M. R. Major , V. W. Wong , E. R. Nelson , M. T. Longaker , G. C. Gurtner , Plast. Reconstr. Surg. 2015, 135, 1489. 25919260 10.1097/PRS.0000000000001193

[25] R. Chen , A. Canales , P. Anikeeva , Nat. Rev. Mater. 2017, 2, 16093. 31448131 10.1038/natrevmats.2016.93 PMC6707077

[26] S. Ramakrishna , L. Tian , C. Wang , S. Liao , W. E. Teo , Med. Devices 2015, 137.

[27] Y. H. Joung , Int. Neurourol. J. 2013, 17, 98. 24143287 10.5213/inj.2013.17.3.98 PMC3797898

[28] J. Rogers , Z. Bao , T. W. Lee , Acc. Chem. Res. 2019, 52, 521. 30884949 10.1021/acs.accounts.9b00048

[29] R. C. Webb , A. P. Bonifas , A. Behnaz , Y. Zhang , K. J. Yu , H. Cheng , M. Shi , Z. Bian , Z. Liu , Y. S. Kim , W. H. Yeo , J. S. Park , J. Song , Y. Li , Y. Huang , A. M. Gorbach , J. A. Rogers , Nat. Mater. 2013, 12, 938. 24037122 10.1038/nmat3755 PMC3825211

[30] C. Larson , B. Peele , S. Li , S. Robinson , M. Totaro , L. Beccai , B. Mazzolai , R. Shepherd , Science 2016, 351, 1071. 26941316 10.1126/science.aac5082

[31] J. Shi , L. Wang , Z. Dai , L. Zhao , M. Du , H. Li , Y. Fang , Small 2018, 14, 1800819. 10.1002/smll.201800819 29847706

[32] H. U. Chung , B. H. Kim , J. Y. Lee , J. Lee , Z. Xie , E. M. Ibler , K. Lee , A. Banks , J. Y. Jeong , J. Kim , C. Ogle , D. Grande , Y. Yu , H. Jang , P. Assem , D. Ryu , J. W. Kwak , M. Namkoong , J. B. Park , Y. Lee , D. H. Kim , A. Ryu , J. Jeong , K. You , B. Ji , Z. Liu , Q. Huo , X. Feng , Y. Deng , Y. Xu , et al., Science 2019, 363, eaau0780.30819934

[33] H. Jeong , L. Wang , T. Ha , R. Mitbander , X. Yang , Z. Dai , S. Qiao , L. Shen , N. Sun , N. Lu , Adv. Mater. Technol. 2019, 4, 1900117.

[34] C. Wang , B. Qi , M. Lin , Z. Zhang , M. Makihata , B. Liu , S. Zhou , Y. H. Huang , H. Hu , Y. Gu , Y. Chen , Y. Lei , T. Lee , S. Chien , K. I. Jang , E. B. Kistler , S. Xu , Nat. Biomed. Eng. 2021, 5, 749. 34272524 10.1038/s41551-021-00763-4

[35] K. Nan , S. D. Kang , K. Li , K. J. Yu , F. Zhu , J. Wang , A. C. Dunn , C. Zhou , Z. Xie , M. T. Agne , H. Wang , H. Luan , Y. Zhang , Y. Huang , G. J. Snyder , J. A. Rogers , Sci. Adv. 2018, 4, eaau5849. 30406207 10.1126/sciadv.aau5849 PMC6214638

[36] S. Choi , J. Park , W. Hyun , J. Kim , J. Kim , Y. B. Lee , C. Song , H. J. Hwang , J. H. Kim , T. Hyeon , D.‐H. Kim , ACS Nano 2015, 9, 6626. 26027637 10.1021/acsnano.5b02790

[37] Z. Huang , Y. Hao , Y. Li , H. Hu , C. Wang , A. Nomoto , T. Pan , Y. Gu , Y. Chen , T. Zhang , W. Li , Y. Lei , N. Kim , C. Wang , L. Zhang , J. W. Ward , A. Maralani , X. Li , M. F. Durstock , A. Pisano , Y. Lin , S. Xu , Nat. Electron. 2018, 1, 473.

[38] J. Kim , H. J. Shim , J. Yang , M. K. Choi , D. C. Kim , J. Kim , T. Hyeon , D. H. Kim , Adv. Mater. 2017, 29, 1700217. 10.1002/adma.201700217 28833644

[39] C.‐H. Chiang , S. M. Won , A. L. Orsborn , K. J. Yu , M. Trumpis , B. Bent , C. Wang , Y. Xue , S. Min , V. Woods , C. Yu , B. H. Kim , S. B. Kim , R. Huq , J. Li , K. J. Seo , F. Vitale , A. Richardson , H. Fang , Y. Huang , K. Shepard , B. Pesaran , J. A. Rogers , J. Viventi , Sci. Transl. Med. 2020, 12, eaay4682. 32269166 10.1126/scitranslmed.aay4682 PMC7478122

[40] E. Song , C. H. Chiang , R. Li , X. Jin , J. Zhao , M. Hill , Y. Xia , L. Li , Y. Huang , S. M. Won , K. J. Yu , X. Sheng , H. Fang , M. A. Alam , Y. Huang , J. Viventi , J. K. Chang , J. A. Rogers , Proc. Natl. Acad. Sci. USA 2019, 116, 15398. 31308234 10.1073/pnas.1907697116 PMC6681732

[41] S. K. Kang , R. K. Murphy , S. W. Hwang , S. M. Lee , D. V. Harburg , N. A. Krueger , J. Shin , P. Gamble , H. Cheng , S. Yu , Z. Liu , J. G. McCall , M. Stephen , H. Ying , J. Kim , G. Park , R. C. Webb , C. H. Lee , S. Chung , D. S. Wie , A. D. Gujar , B. Vemulapalli , A. H. Kim , K. M. Lee , J. Cheng , Y. Huang , S. H. Lee , P. V. Braun , W. Z. Ray , J. A. Rogers , Nature 2016, 530, 71. 26779949 10.1038/nature16492

[42] K. I. Song , H. Seo , D. Seong , S. Kim , K. J. Yu , Y. C. Kim , J. Kim , S. J. Kwon , H. S. Han , I. Youn , H. Lee , D. Son , Nat. Commun. 2020, 11, 4195. 32826916 10.1038/s41467-020-18025-3 PMC7442836

[43] A. D. Mickle , S. M. Won , K. N. Noh , J. Yoon , K. W. Meacham , Y. Xue , L. A. McIlvried , B. A. Copits , V. K. Samineni , K. E. Crawford , D. H. Kim , P. Srivastava , B. H. Kim , S. Min , Y. Shiuan , Y. Yun , M. A. Payne , J. Zhang , H. Jang , Y. Li , H. H. Lai , Y. Huang , S. I. Park , R. W. t. Gereau , J. A. Rogers , Nature 2019, 565, 361. 30602791 10.1038/s41586-018-0823-6 PMC6336505

[44] R. Dong , L. Wang , C. Hang , Z. Chen , X. Liu , L. Zhong , J. Qi , Y. Huang , S. Liu , L. Wang , Y. Lu , X. Jiang , Small 2021, 17, e2006612. 33711201 10.1002/smll.202006612

[45] S. Lienemann , J. Zötterman , S. Farnebo , K. Tybrandt , J. Neural Eng. 2021, 18, 045007. 10.1088/1741-2552/abfebb 33957608

[46] D. Son , J. Kang , O. Vardoulis , Y. Kim , N. Matsuhisa , J. Y. Oh , J. W. To , J. Mun , T. Katsumata , Y. Liu , A. F. McGuire , M. Krason , F. Molina‐Lopez , J. Ham , U. Kraft , Y. Lee , Y. Yun , J. B. Tok , Z. Bao , Nat. Nanotechnol. 2018, 13, 1057. 30127474 10.1038/s41565-018-0244-6

[47] S. Wang , J. Xu , W. Wang , G. N. Wang , R. Rastak , F. Molina‐Lopez , J. W. Chung , S. Niu , V. R. Feig , J. Lopez , T. Lei , S. K. Kwon , Y. Kim , A. M. Foudeh , A. Ehrlich , A. Gasperini , Y. Yun , B. Murmann , J. B. Tok , Z. Bao , Nature 2018, 555, 83. 29466334 10.1038/nature25494

[48] Y. Xu , B. Sun , Y. Ling , Q. Fei , Z. Chen , X. Li , P. Guo , N. Jeon , S. Goswami , Y. Liao , S. Ding , Q. Yu , J. Lin , G. Huang , Z. Yan , Proc. Natl. Acad. Sci. USA 2020, 117, 205. 31871158 10.1073/pnas.1917762116 PMC6955345

[49] H. Tao , S. W. Hwang , B. Marelli , B. An , J. E. Moreau , M. Yang , M. A. Brenckle , S. Kim , D. L. Kaplan , J. A. Rogers , F. G. Omenetto , Proc. Natl. Acad. Sci. USA 2014, 111, 17385. 25422476 10.1073/pnas.1407743111 PMC4267401

[50] D.‐H. Kim , N. Lu , R. Ma , Y.‐S. Kim , R.‐H. Kim , S. Wang , J. Wu , S. M. Won , H. Tao , A. Islam , K. J. Yu , T.‐i. Kim , R. Chowdhury , M. Ying , L. Xu , M. Li , H.‐J. Chung , H. Keum , M. McCormick , P. Liu , Y.‐W. Zhang , F. G. Omenetto , Y. Huang , T. Coleman , J. A. Rogers , Science 2011, 333, 838. 21836009 10.1126/science.1206157

[51] E. D. Głowacki , E. Stavrinidou , D. Khodagholy , Adv. Mater. Technol. 2020, 5, 2000106.

[52] P. Li , H. P. Anwar Ali , W. Cheng , J. Yang , B. C. K. Tee , Adv. Mater. Technol. 2020, 5, 1900856.

[53] J. W. Jeong , M. K. Kim , H. Cheng , W. H. Yeo , X. Huang , Y. Liu , Y. Zhang , Y. Huang , J. A. Rogers , Adv. Healthcare Mater. 2014, 3, 642. 10.1002/adhm.201300334 24132942

[54] P. Chen , X. Sun , H. Peng , Adv. Funct. Mater. 2020, 30, 2001827.

[55] D. Gao , K. Parida , P. S. Lee , Adv. Funct. Mater. 2019, 30, 1907184.

[56] Y. Yu , H. Y. Y. Nyein , W. Gao , A. Javey , Adv. Mater. 2020, 32, 1902083. 10.1002/adma.201902083 31432573

[57] E. Song , J. Li , J. A. Rogers , APL Mater. 2019, 7, 050902.

[58] W. H. Yeo , Y. S. Kim , J. Lee , A. Ameen , L. Shi , M. Li , S. Wang , R. Ma , S. H. Jin , Z. Kang , Y. Huang , J. A. Rogers , Adv. Mater. 2013, 25, 2773. 23440975 10.1002/adma.201204426

[59] M. J. Cima , Nat. Biotechnol. 2014, 32, 642. 25004232 10.1038/nbt.2952

[60] J. Lee , B. Llerena Zambrano , J. Woo , K. Yoon , T. Lee , Adv. Mater. 2020, 32, 1902532. 10.1002/adma.201902532 31495991

[61] J. Tu , R. M. Torrente‐Rodríguez , M. Wang , W. Gao , Adv. Funct. Mater. 2019, 30, 1906713.

[62] P. Jastrzebska‐Perfect , S. Chowdhury , G. D. Spyropoulos , Z. Zhao , C. Cea , J. N. Gelinas , D. Khodagholy , Adv. Funct. Mater. 2020, 30, 1909165.

[63] J. Kim , A. S. Campbell , B. E. de Avila , J. Wang , Nat. Biotechnol. 2019, 37, 389. 30804534 10.1038/s41587-019-0045-y PMC8183422

[64] K. Kang , Y. Cho , K. J. Yu , Micromachines 2018, 9, 263. 10.3390/mi9060263 PMC6187536 30424196

[65] C. Wang , K. Xia , H. Wang , X. Liang , Z. Yin , Y. Zhang , Adv. Mater. 2019, 31, 1801072. 10.1002/adma.201801072 30300444

[66] H. R. Lim , H. S. Kim , R. Qazi , Y. T. Kwon , J. W. Jeong , W. H. Yeo , Adv. Mater. 2020, 32, 1901924. 10.1002/adma.201901924 31282063

[67] S. M. A. Iqbal , I. Mahgoub , E. Du , M. A. Leavitt , W. Asghar , npj Flexible Electron. 2021, 5, 9.

[68] F. Zhang , Y. Zang , D. Huang , C. A. Di , D. Zhu , Nat. Commun. 2015, 6, 8356. 26387591 10.1038/ncomms9356 PMC4595753

[69] A. S. Almuslem , S. F. Shaikh , M. M. Hussain , Adv. Mater. Technol. 2019, 4.

[70] S. Huang , Y. Liu , Y. Zhao , Z. Ren , C. F. Guo , Adv. Funct. Mater. 2018, 29, 1805924.

[71] Y. Ma , Y. Zhang , S. Cai , Z. Han , X. Liu , F. Wang , Y. Cao , Z. Wang , H. Li , Y. Chen , X. Feng , Adv. Mater. 2020, 32, 1902062. 10.1002/adma.201902062 31243834

[72] Y. Gao , L. Yu , J. C. Yeo , C. T. Lim , Adv. Mater. 2020, 32, 1902133. 10.1002/adma.201902133 31339200

[73] Y. Chen , Y. Zhang , Z. Liang , Y. Cao , Z. Han , X. Feng , npj Flexible Electron. 2020, 4, 2.

[74] X. Wang , Z. Liu , T. Zhang , Small 2017, 13, 1602790. 10.1002/smll.201602790 28306196

[75] H. J. Shim , S. H. Sunwoo , Y. Kim , J. H. Koo , D. H. Kim , Adv. Healthcare Mater. 2021, 10, 2002105. 10.1002/adhm.202002105 33506654

[76] Y. Liu , M. Pharr , G. A. Salvatore , ACS Nano 2017, 11, 9614. 28901746 10.1021/acsnano.7b04898

[77] Y. G. Park , G. Y. Lee , J. Jang , S. M. Yun , E. Kim , J. U. Park , Adv. Healthcare Mater. 2021, 10, 2002280.

[78] H. Wu , G. Yang , K. Zhu , S. Liu , W. Guo , Z. Jiang , Z. Li , Adv. Sci. 2021, 8, 2001938. 10.1002/advs.202001938 PMC7816724 33511003

[79] X. Yu , W. Shou , B. K. Mahajan , X. Huang , H. Pan , Adv. Mater. 2018, 30, 1707624. 10.1002/adma.201707624 29736971

[80] S. Choi , H. Lee , R. Ghaffari , T. Hyeon , D. H. Kim , Adv. Mater. 2016, 28, 4203. 26779680 10.1002/adma.201504150

[81] J. Choi , R. Ghaffari , L. B. Baker , J. A. Rogers , Sci. Adv. 2018, 4, eaar3921. 29487915 10.1126/sciadv.aar3921 PMC5817925

[82] Z. Rao , F. Ershad , A. Almasri , L. Gonzalez , X. Wu , C. Yu , Adv. Mater. Technol. 2020, 5, 2000233.

[83] L. Wang , K. Jiang , G. Shen , Adv. Mater. Technol. 2021, 6, 2100107.

[84] S. M. Mirvakili , R. Langer , Nat. Electron. 2021, 4, 464.

[85] Y. S. Oh , J. H. Kim , Z. Xie , S. Cho , H. Han , S. W. Jeon , M. Park , M. Namkoong , R. Avila , Z. Song , S. U. Lee , K. Ko , J. Lee , J. S. Lee , W. G. Min , B. J. Lee , M. Choi , H. U. Chung , J. Kim , M. Han , J. Koo , Y. S. Choi , S. S. Kwak , S. B. Kim , J. Kim , J. Choi , C. M. Kang , J. U. Kim , K. Kwon , S. M. Won , et al., Nat. Commun. 2021, 12, 5008. 34429436 10.1038/s41467-021-25324-w PMC8385057

[86] N. T. Tien , S. Jeon , D. I. Kim , T. Q. Trung , M. Jang , B. U. Hwang , K. E. Byun , J. Bae , E. Lee , J. B. Tok , Z. Bao , N. E. Lee , J. J. Park , Adv. Mater. 2014, 26, 796. 24493054 10.1002/adma.201302869

[87] Z. Yan , L. Wang , Y. Xia , R. Qiu , W. Liu , M. Wu , Y. Zhu , S. Zhu , C. Jia , M. Zhu , R. Cao , Z. Li , X. Wang , Adv. Funct. Mater. 2021, 31, 2100709.

[88] K. Kim , J. Choi , Y. Jeong , I. Cho , M. Kim , S. Kim , Y. Oh , I. Park , Adv. Healthcare Mater. 2019, 8, 1900978. 10.1002/adhm.201900978 31596545

[89] T. Q. Trung , S. Ramasundaram , B. U. Hwang , N. E. Lee , Adv. Mater. 2016, 28, 502. 26607674 10.1002/adma.201504441

[90] S. Gong , L. W. Yap , B. Zhu , Q. Zhai , Y. Liu , Q. Lyu , K. Wang , M. Yang , Y. Ling , D. T. H. Lai , F. Marzbanrad , W. Cheng , Adv. Mater. 2019, 31, 1903789. 10.1002/adma.201903789 31448484

[91] C. Wang , X. Li , H. Hu , L. Zhang , Z. Huang , M. Lin , Z. Zhang , Z. Yin , B. Huang , H. Gong , S. Bhaskaran , Y. Gu , M. Makihata , Y. Guo , Y. Lei , Y. Chen , C. Wang , Y. Li , T. Zhang , Z. Chen , A. P. Pisano , L. Zhang , Q. Zhou , S. Xu , Nat. Biomed. Eng. 2018, 2, 687. 30906648 10.1038/s41551-018-0287-x PMC6428206

[92] W. Jin , E. H. Kim , S. Lee , S. Yu , H. Han , G. Kim , S. W. Lee , J. Jang , C. E. Lee , W. Shim , C. Park , Adv. Funct. Mater. 2021, 31, 2010492.

[93] Y. Chen , B. Lu , Y. Chen , X. Feng , Sci. Rep. 2015, 5, 11505. 26095941 10.1038/srep11505 PMC4476093

[94] R. C. Webb , Y. Ma , S. Krishnan , Y. Li , S. Yoon , X. Guo , X. Feng , Y. Shi , M. Seidel , N. H. Cho , J. Kurniawan , J. Ahad , N. Sheth , J. Kim , J. G. T. VI , T. Darlington , K. Chang , W. Huang , J. Ayers , A. Gruebele , R. M. Pielak , M. J. Slepian , Y. Huang , A. M. Gorbach , J. A. Rogers , Sci. Adv. 2015, 1, e1500701. 26601309 10.1126/sciadv.1500701 PMC4646823

[95] C. Y. Lee , S. J. Lee , M. S. Tang , P. C. Chen , Sensors (Basel) 2011, 11, 9942. 22163735 10.3390/s111009942 PMC3231273

[96] Y. Q. Li , W. B. Zhu , X. G. Yu , P. Huang , S. Y. Fu , N. Hu , K. Liao , ACS Appl. Mater. Interfaces 2016, 8, 33189. 27934197 10.1021/acsami.6b11196

[97] C. Zhu , A. Chortos , Y. Wang , R. Pfattner , T. Lei , A. C. Hinckley , I. Pochorovski , X. Yan , J. W. F. To , J. Y. Oh , J. B. H. Tok , Z. Bao , B. Murmann , Nat. Electron. 2018, 1, 183.

[98] M. Sang , K. Kang , Y. Zhang , H. Zhang , K. Kim , M. Cho , J. Shin , J. H. Hong , T. Kim , S. K. Lee , W. H. Yeo , J. W. Lee , T. Lee , B. Xu , K. J. Yu , Adv. Mater. 2022, 34, 2105865. 10.1002/adma.202105865 34750868

[99] Y. Lu , Y. Fujita , S. Honda , S. H. Yang , Y. Xuan , K. Xu , T. Arie , S. Akita , K. Takei , Adv. Healthcare Mater. 2021, 10, 2100103. 10.1002/adhm.202100103 33955182

[100] J. Shin , B. Jeong , J. Kim , V. B. Nam , Y. Yoon , J. Jung , S. Hong , H. Lee , H. Eom , J. Yeo , J. Choi , D. Lee , S. H. Ko , Adv. Mater. 2020, 32, 1905527. 10.1002/adma.201905527 31696977

[101] P. Won , S. Jeong , C. Majidi , S. H. Ko , iScience 2021, 24, 102698. 34195573 10.1016/j.isci.2021.102698 PMC8239807

[102] Y. Yoon , P. L. Truong , D. Lee , S. H. Ko , ACS Nanosci. Au 2022, 2, 64. 10.1021/acsnanoscienceau.1c00029 PMC10114907 37101661

[103] K. K. Kim , I. Ha , M. Kim , J. Choi , P. Won , S. Jo , S. H. Ko , Nat. Commun. 2020, 11, 2149. 32358525 10.1038/s41467-020-16040-y PMC7195472

[104] K. Kang , J. Park , K. Kim , K. J. Yu , Nano Res. 2021, 14, 3096.

[105] C. M. Lochner , Y. Khan , A. Pierre , A. C. Arias , Nat. Commun. 2014, 5, 5745. 25494220 10.1038/ncomms6745

[106] C. Hou , Z. Xu , W. Qiu , R. Wu , Y. Wang , Q. Xu , X. Y. Liu , W. Guo , Small 2019, 15, 1805084. 10.1002/smll.201805084 30690886

[107] T. Ha , J. Tran , S. Liu , H. Jang , H. Jeong , R. Mitbander , H. Huh , Y. Qiu , J. Duong , R. L. Wang , P. Wang , A. Tandon , J. Sirohi , N. Lu , Adv. Sci. 2019, 6, 1900290. 10.1002/advs.201900290 PMC6662084 31380208

[108] M. Pal , A. Giri , D. W. Kim , S. Shin , M. Kong , K. Thiyagarajan , J. Kwak , O. F. N. Okello , S. Y. Choi , U. Jeong , ACS Nano 2019, 13, 7175. 31149801 10.1021/acsnano.9b02649

[109] D. Han , Y. Khan , J. Ting , S. M. King , N. Yaacobi‐Gross , M. J. Humphries , C. J. Newsome , A. C. Arias , Adv. Mater. 2017, 29, 1606206. 10.1002/adma.201606206 28394455

[110] Y. Khan , D. Han , A. Pierre , J. Ting , X. Wang , C. M. Lochner , G. Bovo , N. Yaacobi‐Gross , C. Newsome , R. Wilson , A. C. Arias , Proc. Natl. Acad. Sci. USA 2018, 115, E11015. 30404911 10.1073/pnas.1813053115 PMC6255203

[111] S. Choi , S. I. Han , D. Jung , H. J. Hwang , C. Lim , S. Bae , O. K. Park , C. M. Tschabrunn , M. Lee , S. Y. Bae , J. W. Yu , J. H. Ryu , S. W. Lee , K. Park , P. M. Kang , W. B. Lee , R. Nezafat , T. Hyeon , D. H. Kim , Nat. Nanotechnol. 2018, 13, 1048. 30104619 10.1038/s41565-018-0226-8

[112] D. Y. Park , D. J. Joe , D. H. Kim , H. Park , J. H. Han , C. K. Jeong , H. Park , J. G. Park , B. Joung , K. J. Lee , Adv. Mater. 2017, 29. 10.1002/adma.201702308 28714239

[113] F. Stauffer , M. Thielen , C. Sauter , S. Chardonnens , S. Bachmann , K. Tybrandt , C. Peters , C. Hierold , J. Voros , Adv. Healthcare Mater. 2018, 7, 1700994. 10.1002/adhm.201700994 29330962

[114] S. M. Lee , J. H. Lee , S. Lee , in 2015 IEEE 15th International Conference on Nanotechnology (IEEE‐NANO), IEEE, Rome, Italy 2015, p. 2015.

[115] J. J. Norton , D. S. Lee , J. W. Lee , W. Lee , O. Kwon , P. Won , S. Y. Jung , H. Cheng , J. W. Jeong , A. Akce , S. Umunna , I. Na , Y. H. Kwon , X. Q. Wang , Z. Liu , U. Paik , Y. Huang , T. Bretl , W. H. Yeo , J. A. Rogers , Proc. Natl. Acad. Sci. USA 2015, 112, 3920. 25775550 10.1073/pnas.1424875112 PMC4386388

[116] D. Kim , J. Bang , P. Won , Y. Kim , J. Jung , J. Lee , J. Kwon , H. Lee , S. Hong , N. L. Jeon , S. Han , S. H. Ko , Adv. Mater. Technol. 2020, 5, 2000661.

[117] K. K. Kim , J. Choi , J.‐H. Kim , S. Nam , S. H. Ko , Adv. Funct. Mater. 2022, 32, 2106329.

[118] H. Jeong , L. Wang , T. Ha , R. Mitbander , X. Yang , Z. Dai , S. Qiao , L. Shen , N. Sun , N. Lu , Adv. Mater. Technol. 2019, 4, 1900117.

[119] S. Song , K. Y. Kim , S. H. Lee , K. K. Kim , K. Lee , W. Lee , H. Jeon , S. H. Ko , Adv. NanoBiomed Res. 2022, 2, 2100111.

[120] K. K. Kim , Y. Suh , S. H. Ko , Adv. Intell. Syst. 2021, 3, 2000157.

[121] H. Yao , A. J. Shum , M. Cowan , I. Lahdesmaki , B. A. Parviz , Biosens. Bioelectron. 2011, 26, 3290. 21257302 10.1016/j.bios.2010.12.042 PMC3043144

[122] S. Iguchi , H. Kudo , T. Saito , M. Ogawa , H. Saito , K. Otsuka , A. Funakubo , K. Mitsubayashi , Biomed. Microdevices 2007, 9, 603. 17520370 10.1007/s10544-007-9073-3

[123] H. Lee , T. K. Choi , Y. B. Lee , H. R. Cho , R. Ghaffari , L. Wang , H. J. Choi , T. D. Chung , N. Lu , T. Hyeon , S. H. Choi , D. H. Kim , Nat. Nanotechnol. 2016, 11, 566. 26999482 10.1038/nnano.2016.38

[124] H. Lin , J. Tan , J. Zhu , S. Lin , Y. Zhao , W. Yu , H. Hojaiji , B. Wang , S. Yang , X. Cheng , Z. Wang , E. Tang , C. Yeung , S. Emaminejad , Nat. Commun. 2020, 11, 4405. 32879320 10.1038/s41467-020-18238-6 PMC7467936

[125] A. Koh , D. Kang , Y. Xue , S. Lee , R. M. Pielak , J. Kim , T. Hwang , S. Min , A. Banks , P. Bastien , M. C. Manco , L. Wang , K. R. Ammann , K.‐I. Jang , P. Won , S. Han , R. Ghaffari , U. Paik , M. J. Slepian , G. Balooch , Y. Huang , J. A. Rogers , Sci. Transl. Med. 2016, 8, 366ra165. 10.1126/scitranslmed.aaf2593 PMC5429097 27881826

[126] S. Imani , A. J. Bandodkar , A. M. Mohan , R. Kumar , S. Yu , J. Wang , P. P. Mercier , Nat. Commun. 2016, 7, 11650. 27212140 10.1038/ncomms11650 PMC4879240

[127] K. Kwon , J. U. Kim , Y. Deng , S. R. Krishnan , J. Choi , H. Jang , K. Lee , C.‐J. Su , I. Yoo , Y. Wu , L. Lipschultz , J.‐H. Kim , T. S. Chung , D. Wu , Y. Park , T.‐i. Kim , R. Ghaffari , S. Lee , Y. Huang , J. A. Rogers , Nat. Electron. 2021, 4, 302.

[128] S. Emaminejad , W. Gao , E. Wu , Z. A. Davies , H. Yin Yin Nyein , S. Challa , S. P. Ryan , H. M. Fahad , K. Chen , Z. Shahpar , S. Talebi , C. Milla , A. Javey , R. W. Davis , Proc. Natl. Acad. Sci. USA 2017, 114, 4625. 28416667 10.1073/pnas.1701740114 PMC5422810

[129] M. J. Tierney , J. A. Tamada , R. O. Potts , L. Jovanovic , S. Garg , Biosens. Bioelectron. 2001, 16, 621. 11679237 10.1016/s0956-5663(01)00189-0

[130] W. Gao , S. Emaminejad , H. Y. Y. Nyein , S. Challa , K. Chen , A. Peck , H. M. Fahad , H. Ota , H. Shiraki , D. Kiriya , D. H. Lien , G. A. Brooks , R. W. Davis , A. Javey , Nature 2016, 529, 509. 26819044 10.1038/nature16521 PMC4996079

[131] M. S. Mannoor , H. Tao , J. D. Clayton , A. Sengupta , D. L. Kaplan , R. R. Naik , N. Verma , F. G. Omenetto , M. C. McAlpine , Nat. Commun. 2012, 3, 763. 22453836 10.1038/ncomms1767

[132] D. Lee , T. Cui , Biosens. Bioelectron. 2010, 25, 2259. 20417088 10.1016/j.bios.2010.03.003

[133] R. C. Reid , S. R. Jones , D. P. Hickey , S. D. Minteer , B. K. Gale , Electrochim. Acta 2016, 203, 30.

[134] J. Kim , G. Valdes‐Ramirez , A. J. Bandodkar , W. Jia , A. G. Martinez , J. Ramirez , P. Mercier , J. Wang , Analyst 2014, 139, 1632. 24496180 10.1039/c3an02359a

[135] L. Lipani , B. G. R. Dupont , F. Doungmene , F. Marken , R. M. Tyrrell , R. H. Guy , A. Ilie , Nat. Nanotechnol. 2018, 13, 504. 29632401 10.1038/s41565-018-0112-4

[136] J. Kim , I. Jeerapan , S. Imani , T. N. Cho , A. Bandodkar , S. Cinti , P. P. Mercier , J. Wang , ACS Sens. 2016, 1, 1011.

[137] D. Kinnamon , R. Ghanta , K. C. Lin , S. Muthukumar , S. Prasad , Sci. Rep. 2017, 7, 13312. 29042582 10.1038/s41598-017-13684-7 PMC5645384

[138] J. Choi , Y. Xue , W. Xia , T. R. Ray , J. T. Reeder , A. J. Bandodkar , D. Kang , S. Xu , Y. Huang , J. A. Rogers , Lab Chip 2017, 17, 2572. 28664954 10.1039/c7lc00525c PMC5561737

[139] J. Park , J. Kim , S.‐Y. Kim , W. H. Cheong , J. Jang , Y.‐G. Park , K. Na , Y.‐T. Kim , J. H. Heo , C. Y. Lee , J. H. Lee , F. Bien , J.‐U. Park , Sci. Adv. 2018, 4, eaap9841. 29387797 10.1126/sciadv.aap9841 PMC5787380

[140] W. C. Mak , K. Y. Cheung , J. Orban , C. J. Lee , A. P. Turner , M. Griffith , ACS Appl. Mater. Interfaces 2015, 7, 25487. 26512953 10.1021/acsami.5b08644

[141] J. Choi , D. Kang , S. Han , S. B. Kim , J. A. Rogers , Adv. Healthcare Mater. 2017, 6, 1601355. 10.1002/adhm.201601355 28105745

[142] J. T. Reeder , J. Choi , Y. Xue , P. Gutruf , J. Hanson , M. Liu , T. Ray , A. J. Bandodkar , R. Avila , W. Xia , S. Krishnan , S. Xu , K. Barnes , M. Pahnke , R. Ghaffari , Y. Huang , J. A. Rogers , Sci. Adv. 2019, 5, eaau6356. 30746456 10.1126/sciadv.aau6356 PMC6357724

[143] M. Elsherif , M. U. Hassan , A. K. Yetisen , H. Butt , ACS Nano 2018, 12, 5452. 29750502 10.1021/acsnano.8b00829 PMC6107296

[144] J. Kim , W. R. de Araujo , I. A. Samek , A. J. Bandodkar , W. Jia , B. Brunetti , T. R. L. C. Paixão , J. Wang , Electrochem. Commun. 2015, 51, 41.

[145] H. Lee , C. Song , Y. S. Hong , M. S. Kim , H. R. Cho , T. Kang , K. Shin , S. H. Choi , T. Hyeon , D.‐H. Kim , Sci. Adv. 2017, 3, e1601314. 28345030 10.1126/sciadv.1601314 PMC5342654

[146] D. H. Keum , S.‐K. Kim , J. Koo , G.‐H. Lee , C. Jeon , J. W. Mok , B. H. Mun , K. J. Lee , E. Kamrani , C.‐K. Joo , S. Shin , J.‐Y. Sim , D. Myung , S. H. Yun , Z. Bao , S. K. Hahn , Sci. Adv. 2020, 6, eaba3252. 32426469 10.1126/sciadv.aba3252 PMC7182412

[147] Y. Lee , C. Howe , S. Mishra , D. S. Lee , M. Mahmood , M. Piper , Y. Kim , K. Tieu , H. S. Byun , J. P. Coffey , M. Shayan , Y. Chun , R. M. Costanzo , W. H. Yeo , Proc. Natl. Acad. Sci. USA 2018, 115, 5377. 29735689 10.1073/pnas.1719573115 PMC6003521

[148] K. K. Kim , J. Choi , S. H. Ko , Adv. Healthcare Mater. 2021, 10, 2002286.

[149] C. Dagdeviren , Y. Su , P. Joe , R. Yona , Y. Liu , Y. S. Kim , Y. Huang , A. R. Damadoran , J. Xia , L. W. Martin , Y. Huang , J. A. Rogers , Nat. Commun. 2014, 5, 4496. 25092496 10.1038/ncomms5496

[150] Y. Liu , J. J. S. Norton , R. Qazi , Z. Zou , K. R. Ammann , H. Liu , L. Yan , P. L. Tran , K.‐I. Jang , J. W. Lee , D. Zhang , K. A. Kilian , S. H. Jung , T. Bretl , J. Xiao , M. J. Slepian , Y. Huang , J.‐W. Jeong , J. A. Rogers , Sci. Adv. 2016, 2, e1601185. 28138529 10.1126/sciadv.1601185 PMC5262452

[151] H. S. Lee , J. Chung , G.‐T. Hwang , C. K. Jeong , Y. Jung , J.‐H. Kwak , H. Kang , M. Byun , W. D. Kim , S. Hur , S.‐H. Oh , K. J. Lee , Adv. Funct. Mater. 2014, 24, 6914.

[152] C. Lang , J. Fang , H. Shao , X. Ding , T. Lin , Nat. Commun. 2016, 7, 11108. 27005010 10.1038/ncomms11108 PMC4814578

[153] L. Q. Tao , H. Tian , Y. Liu , Z. Y. Ju , Y. Pang , Y. Q. Chen , D. Y. Wang , X. G. Tian , J. C. Yan , N. Q. Deng , Y. Yang , T. L. Ren , Nat. Commun. 2017, 8, 14579. 28232739 10.1038/ncomms14579 PMC5333117

[154] X. Fan , J. Chen , J. Yang , P. Bai , Z. Li , Z. L. Wang , ACS Nano 2015, 9, 4236. 25790372 10.1021/acsnano.5b00618

[155] H. Li , Y. Ding , H. Ha , Y. Shi , L. Peng , X. Zhang , C. J. Ellison , G. Yu , Adv. Mater. 2017, 29, 1700898. 10.1002/adma.201700898 28387425

[156] J. Liang , L. Li , X. Niu , Z. Yu , Q. Pei , Nat. Photonics 2013, 7, 817.

[157] S. R. Madhvapathy , Y. Ma , M. Patel , S. Krishnan , C. Wei , Y. Li , S. Xu , X. Feng , Y. Huang , J. A. Rogers , Adv. Funct. Mater. 2018, 28, 1802083.

[158] X. Wang , Y. Zhang , X. Zhang , Z. Huo , X. Li , M. Que , Z. Peng , H. Wang , C. Pan , Adv. Mater. 2018, 30, 1706738. 10.1002/adma.201706738 29411908

[159] C. K. Jeong , J. Lee , S. Han , J. Ryu , G. T. Hwang , D. Y. Park , J. H. Park , S. S. Lee , M. Byun , S. H. Ko , K. J. Lee , Adv. Mater. 2015, 27, 2866. 25824939 10.1002/adma.201500367

[160] J. Liang , L. Li , K. Tong , Z. Ren , W. Hu , X. Niu , Y. Chen , Q. Pei , ACS Nano 2014, 8, 1590. 24471886 10.1021/nn405887k

[161] X. Chen , K. Parida , J. Wang , J. Xiong , M. F. Lin , J. Shao , P. S. Lee , ACS Appl. Mater. Interfaces 2017, 9, 42200. 29111642 10.1021/acsami.7b13767

[162] M. A. Gonzalez‐Gonzalez , A. Kanneganti , A. Joshi‐Imre , A. G. Hernandez‐Reynoso , G. Bendale , R. Modi , M. Ecker , A. Khurram , S. F. Cogan , W. E. Voit , M. I. Romero‐Ortega , Sci. Rep. 2018, 8, 16390. 30401906 10.1038/s41598-018-34566-6 PMC6219541

[163] Y. Li , Y. Ma , C. Wei , H. Luan , S. Xu , M. Han , H. Zhao , C. Liang , Q. Yang , Y. Yang , K. E. Crawford , X. Feng , Y. Huang , J. A. Rogers , Adv. Funct. Mater. 2018, 28, 1801380.

[164] J. H. Koo , S. Jeong , H. J. Shim , D. Son , J. Kim , D. C. Kim , S. Choi , J. I. Hong , D. H. Kim , ACS Nano 2017, 11, 10032. 28837773 10.1021/acsnano.7b04292

[165] S. Park , S. W. Heo , W. Lee , D. Inoue , Z. Jiang , K. Yu , H. Jinno , D. Hashizume , M. Sekino , T. Yokota , K. Fukuda , K. Tajima , T. Someya , Nature 2018, 561, 516. 30258137 10.1038/s41586-018-0536-x

[166] S. Xu , Y. Zhang , J. Cho , J. Lee , X. Huang , L. Jia , J. A. Fan , Y. Su , J. Su , H. Zhang , H. Cheng , B. Lu , C. Yu , C. Chuang , T. I. Kim , T. Song , K. Shigeta , S. Kang , C. Dagdeviren , I. Petrov , P. V. Braun , Y. Huang , U. Paik , J. A. Rogers , Nat. Commun. 2013, 4, 1543. 23443571 10.1038/ncomms2553

[167] K.‐B. Kim , W. Jang , J. Y. Cho , S. B. Woo , D. H. Jeon , J. H. Ahn , S. D. Hong , H. Y. Koo , T. H. Sung , Nano Energy 2018, 54, 91.

[168] Y. Zou , P. Tan , B. Shi , H. Ouyang , D. Jiang , Z. Liu , H. Li , M. Yu , C. Wang , X. Qu , L. Zhao , Y. Fan , Z. L. Wang , Z. Li , Nat. Commun. 2019, 10, 2695. 31217422 10.1038/s41467-019-10433-4 PMC6584498

[169] Y. Jung , J. Choi , Y. Yoon , H. Park , J. Lee , S. H. Ko , Nano Energy 2022, 95, 107002.

[170] H. Qin , L. Xu , S. Lin , F. Zhan , K. Dong , K. Han , H. Wang , Y. Feng , Z. L. Wang , Adv. Funct. Mater. 2022, 32, 2111662.

[171] H. Moon , H. Lee , J. Kwon , Y. D. Suh , D. K. Kim , I. Ha , J. Yeo , S. Hong , S. H. Ko , Sci. Rep. 2017, 7, 41981. 28155913 10.1038/srep41981 PMC5290463

[172] G. Brändén , G. Hammarin , R. Harimoorthy , A. Johansson , D. Arnlund , E. Malmerberg , A. Barty , S. Tångefjord , P. Berntsen , D. P. DePonte , C. Seuring , T. A. White , F. Stellato , R. Bean , K. R. Beyerlein , L. M. G. Chavas , H. Fleckenstein , C. Gati , U. Ghoshdastider , L. Gumprecht , D. Oberthür , D. Popp , M. Seibert , T. Tilp , M. Messerschmidt , G. J. Williams , N. D. Loh , H. N. Chapman , P. Zwart , M. Liang , et al., Nat. Commun. 2019, 10, 2589. 31197138 10.1038/s41467-019-10448-x PMC6565740

[173] S. Hong , H. Lee , J. Lee , J. Kwon , S. Han , Y. D. Suh , H. Cho , J. Shin , J. Yeo , S. H. Ko , Adv. Mater. 2015, 27, 4744. 26177729 10.1002/adma.201500917

[174] H. Lee , S. Hong , J. Lee , Y. D. Suh , J. Kwon , H. Moon , H. Kim , J. Yeo , S. H. Ko , ACS Appl. Mater. Interfaces 2016, 8, 15449. 27285849 10.1021/acsami.6b04364

[175] P. Won , J. J. Park , T. Lee , I. Ha , S. Han , M. Choi , J. Lee , S. Hong , K.‐J. Cho , S. H. Ko , Nano Lett. 2019, 19, 6087. 31411037 10.1021/acs.nanolett.9b02014

[176] L. Chen , X. Chang , H. Wang , J. Chen , Y. Zhu , Nano Energy 2022, 96, 107077.

[177] P. Won , K. K. Kim , H. Kim , J. J. Park , I. Ha , J. Shin , J. Jung , H. Cho , J. Kwon , H. Lee , S. H. Ko , Adv. Mater. 2021, 33, 2002397. 10.1002/adma.202002397 33089569

[178] M. Sang , J. Shin , K. Kim , K. J. Yu , Nanomaterials 2019, 9, 374. 10.3390/nano9030374 PMC6474003 30841599

[179] T. Kim , M. Cho , K. J. Yu , Materials 2018, 11. 10.3390/ma11071163 PMC6073353 29986539

[180] S. H. Ko , J. Rogers , Adv. Funct. Mater. 2021, 31, 2106546.

[181] K. Wang , L. W. Yap , S. Gong , R. Wang , S. J. Wang , W. Cheng , Adv. Funct. Mater. 2021, 31, 2008347.

[182] J. Lee , H. Sul , W. Lee , K. R. Pyun , I. Ha , D. Kim , H. Park , H. Eom , Y. Yoon , J. Jung , D. Lee , S. H. Ko , Adv. Funct. Mater. 2020, 30, 1909171.

[183] J. Lee , D. Kim , H. Sul , S. H. Ko , Adv. Funct. Mater. 2021, 31, 2007376.

[184] M. Mahmood , N. Kim , M. Mahmood , H. Kim , H. Kim , N. Rodeheaver , M. Sang , K. J. Yu , W.‐H. Yeo , Biosens. Bioelectron. 2022, 210, 114333. 35525171 10.1016/j.bios.2022.114333

[185] H. Li , Y. Ma , Y. Huang , Mater. Horiz. 2021, 8, 383. 34821261 10.1039/d0mh00483a

[186] J. Fortin , D. E. Rogge , C. Fellner , D. Flotzinger , J. Grond , K. Lerche , B. Saugel , Nat. Commun. 2021, 12, 1387. 33654082 10.1038/s41467-021-21271-8 PMC7925606

[187] X. Quan , J. Liu , T. Roxlo , S. Siddharth , W. Leong , A. Muir , S. M. Cheong , A. Rao , Sensors (Basel) 2021, 21, 4273. 34206457 10.3390/s21134273 PMC8271585

[188] S. Y. Yoo , J. E. Ahn , G. Cserey , H. Y. Lee , J. M. Seo , Sensors (Basel) 2019, 19. 10.3390/s19071744 PMC6480067 30979050

[189] D. Nachman , K. Constantini , G. Poris , L. Wagnert‐Avraham , S. D. Gertz , R. Littman , E. Kabakov , A. Eisenkraft , Y. Gepner , Sci. Rep. 2020, 10, 17684. 33077774 10.1038/s41598-020-74686-6 PMC7573605

[190] C. Liu , J. T. Kim , S. S. Kwak , A. Hourlier‐Fargette , R. Avila , J. Vogl , A. Tzavelis , H. U. Chung , J. Y. Lee , D. H. Kim , D. Ryu , K. B. Fields , J. L. Ciatti , S. Li , M. Irie , A. Bradley , A. Shukla , J. Chavez , E. C. Dunne , S. S. Kim , J. Kim , J. B. Park , H. H. Jo , J. Kim , M. C. Johnson , J. W. Kwak , S. R. Madhvapathy , S. Xu , C. M. Rand , L. E. Marsillio , et al., Adv. Healthcare Mater. 2021, 10, 2100383.

[191] J. Deng , H. Yuk , J. Wu , C. E. Varela , X. Chen , E. T. Roche , C. F. Guo , X. Zhao , Nat. Mater. 2021, 20, 229. 32989277 10.1038/s41563-020-00814-2

[192] K. Domansky , J. D. Sliz , N. Wen , C. Hinojosa , G. Thompson , J. P. Fraser , T. Hamkins‐Indik , G. A. Hamilton , D. Levner , D. E. Ingber , Microfluid. Nanofluid. 2017, 21, 107.

[193] S. Koosehgol , M. Ebrahimian‐Hosseinabadi , M. Alizadeh , A. Zamanian , Mater. Sci. Eng., C 2017, 79, 66. 10.1016/j.msec.2017.05.001 28629065

[194] J. Wei , M. Liao , A. Ma , Y. Chen , Z. Duan , X. Hou , M. Li , N. Jiang , J. Yu , Compos. Commun. 2020, 17, 141.

[195] B. Zhu , S. Gong , F. Lin , Y. Wang , Y. Ling , T. An , W. Cheng , Adv. Electron. Mater. 2019, 5, 1800509.

[196] M.‐H. Tsai , I.‐H. Tseng , Y.‐C. Huang , H.‐P. Yu , P.‐Y. Chang , Adv. Eng. Mater. 2016, 18, 582.

[197] Q.‐H. Lu , F. Zheng , in Advanced Polyimide Materials (Ed: S.‐Y. Yang ), Elsevier, Amsterdam 2018, p. 195.

[198] W. Ning , Z. Wang , P. Liu , D. Zhou , S. Yang , J. Wang , Q. Li , S. Fan , K. Jiang , Carbon 2018, 139, 1136.

[199] Y. Guo , X. Yang , K. Ruan , J. Kong , M. Dong , J. Zhang , J. Gu , Z. Guo , ACS Appl. Mater. Interfaces 2019, 11, 25465. 31268646 10.1021/acsami.9b10161

[200] J. Fahlteich , W. Schönberger , M. Fahland , N. Schiller , Surf. Coat. Technol. 2011, 205, S141.

[201] K. Gunes , A. I. Isayev , X. Li , C. Wesdemiotis , Polymer 2010, 51, 1071.

[202] C. M. A. Lopes , M. I. Felisberti , Polym. Test. 2004, 23, 637.

[203] R. Xu , J. W. Lee , T. Pan , S. Ma , J. Wang , J. H. Han , Y. Ma , J. A. Rogers , Y. Huang , Adv. Funct. Mater. 2017, 27, 1604545. 29046624 10.1002/adfm.201604545 PMC5642935

[204] Lyondell Basell group companies, ecoflex F Blend C1200, 2013, 1.

[205] I. Vroman , L. Tighzert , Materials 2009, 2, 307.

[206] S. H. Kim , S. Jung , I. S. Yoon , C. Lee , Y. Oh , J.‐M. Hong , Adv. Mater. 2018, 30, 1800109. 10.1002/adma.201800109 29761554

[207] M. D. Borysiak , K. S. Bielawski , N. J. Sniadecki , C. F. Jenkel , B. D. Vogt , J. D. Posner , Lab Chip 2013, 13, 2773. 23670166 10.1039/c3lc50426c PMC3799950

[208] P. Gupta , M. Bera , P. K. Maji , Polym. Adv. Technol. 2017, 28, 1428.

[209] S.‐W. Youn , A. Ueno , M. Takahashi , R. Maeda , Microelectron. Eng. 2008, 85, 1924.

[210] Kayaku Advanced Materials, Inc., SU‐8 2000 Permanent Negative Epoxy Photoresist 2020, 1.

[211] Diamond‐MT, Parylene 101 a complete guide.

[212] N. Coron , C. Cuesta , E. García , C. Ginestra , T. A. Girard , P. de Marcillac , M. Martínez , Y. Ortigoza , A. Ortiz de Solórzano , C. Pobes , J. Puimedón , T. Redon , M. L. Sarsa , L. Torres , P. Valko , J. Á. Villar , EPJ Web Conf. 2014, 65, 02001.

[213] S. P. T. Co., Ltd., Kraton D4150 K Linear Triblock Copolymer Datasheet.

[214] Z. Ma , Q. Huang , Q. Xu , Q. Zhuang , X. Zhao , Y. Yang , H. Qiu , Z. Yang , C. Wang , Y. Chai , Z. Zheng , Nat. Mater. 2021, 20, 859. 33603185 10.1038/s41563-020-00902-3

[215] A. Volkov , Encyclopedia of Membranes, 2013, p. 1.

[216] M. Kheirabadi , R. Bagheri , K. Kabiri , Polym. Bull. 2015, 72, 1663.

[217] Y. You , J. Yang , Q. Zheng , N. Wu , Z. Lv , Z. Jiang , Sci. Rep. 2020, 10, 11727. 32678203 10.1038/s41598-020-68678-9 PMC7366625

[218] D. Lu , Y. Yan , Y. Deng , Q. Yang , J. Zhao , M. H. Seo , W. Bai , M. R. MacEwan , Y. Huang , W. Z. Ray , J. A. Rogers , Adv. Funct. Mater. 2020, 30, 2003754.

[219] S. A. Dual , B. Llerena Zambrano , S. Sundermann , N. Cesarovic , M. Kron , K. Magkoutas , J. Hengsteler , V. Falk , C. Starck , M. Meboldt , J. Voros , M. Schmid Daners , Adv. Healthcare Mater. 2020, 9, 2000855. 10.1002/adhm.202000855 32893478

[220] G. Yao , L. Kang , J. Li , Y. Long , H. Wei , C. A. Ferreira , J. J. Jeffery , Y. Lin , W. Cai , X. Wang , Nat. Commun. 2018, 9, 5349. 30559435 10.1038/s41467-018-07764-z PMC6297229

[221] F. Jin , T. Li , T. Yuan , L. Du , C. Lai , Q. Wu , Y. Zhao , F. Sun , L. Gu , T. Wang , Z. Q. Feng , Adv. Mater. 2021, 33, 2104175. 10.1002/adma.202104175 34608668

[222] C. M. Boutry , Y. Kaizawa , B. C. Schroeder , A. Chortos , A. Legrand , Z. Wang , J. Chang , P. Fox , Z. Bao , Nat. Electron. 2018, 1, 314.

[223] P. Gutruf , R. T. Yin , K. B. Lee , J. Ausra , J. A. Brennan , Y. Qiao , Z. Xie , R. Peralta , O. Talarico , A. Murillo , S. W. Chen , J. P. Leshock , C. R. Haney , E. A. Waters , C. Zhang , H. Luan , Y. Huang , G. Trachiotis , I. R. Efimov , J. A. Rogers , Nat. Commun. 2019, 10, 5742. 31848334 10.1038/s41467-019-13637-w PMC6917818

[224] E. J. Curry , K. Ke , M. T. Chorsi , K. S. Wrobel , A. N. Miller, 3rd , A. Patel , I. Kim , J. Feng , L. Yue , Q. Wu , C. L. Kuo , K. W. Lo , C. T. Laurencin , H. Ilies , P. K. Purohit , T. D. Nguyen , Proc. Natl. Acad. Sci. USA 2018, 115, 909. 29339509 10.1073/pnas.1710874115 PMC5798324

[225] W. Bai , J. Shin , R. Fu , I. Kandela , D. Lu , X. Ni , Y. Park , Z. Liu , T. Hang , D. Wu , Y. Liu , C. R. Haney , I. Stepien , Q. Yang , J. Zhao , K. R. Nandoliya , H. Zhang , X. Sheng , L. Yin , K. MacRenaris , A. Brikha , F. Aird , M. Pezhouh , J. Hornick , W. Zhou , J. A. Rogers , Nat. Biomed. Eng. 2019, 3, 644. 31391594 10.1038/s41551-019-0435-y

[226] H. Zhang , P. Gutruf , K. Meacham , M. C. Montana , X. Zhao , A. M. Chiarelli , A. Vázquez‐Guardado , A. Norris , L. Lu , Q. Guo , C. Xu , Y. Wu , H. Zhao , X. Ning , W. Bai , I. Kandela , C. R. Haney , D. Chanda , R. W. Gereau , J. A. Rogers , Sci. Adv. 2019, 5, eaaw0873. 30873435 10.1126/sciadv.aaw0873 PMC6408152

[227] J. Li , E. Song , C. H. Chiang , K. J. Yu , J. Koo , H. Du , Y. Zhong , M. Hill , C. Wang , J. Zhang , Y. Chen , L. Tian , Y. Zhong , G. Fang , J. Viventi , J. A. Rogers , Proc. Natl. Acad. Sci. USA 2018, 115, E9542. 30228119 10.1073/pnas.1813187115 PMC6187144

[228] J. Shin , Z. Liu , W. Bai , Y. Liu , Y. Yan , Y. Xue , I. Kandela , M. Pezhouh , M. R. MacEwan , Y. Huang , W. Z. Ray , W. Zhou , J. A. Rogers , Sci. Adv. 2019, 5, eaaw1899. 31281889 10.1126/sciadv.aaw1899 PMC6611687

[229] Y. U. Cho , S. L. Lim , J.‐H. Hong , K. J. Yu , npj Flexible Electron. 2022, 6, 53.

[230] Y. U. Cho , J. Y. Lee , U.‐J. Jeong , S. H. Park , S. L. Lim , K. Y. Kim , J. W. Jang , J. H. Park , H. W. Kim , H. Shin , H. Jeon , Y. M. Jung , I.‐J. Cho , K. J. Yu , Adv. Funct. Mater. 2022, 32, 2105568.

[231] Y. Cho , S. Park , J. Lee , K. J. Yu , Adv. Mater. 2021, 33, 2005786. 10.1002/adma.202005786 PMC11468537 34050691

[232] J. Koo , M. R. MacEwan , S. K. Kang , S. M. Won , M. Stephen , P. Gamble , Z. Xie , Y. Yan , Y. Y. Chen , J. Shin , N. Birenbaum , S. Chung , S. B. Kim , J. Khalifeh , D. V. Harburg , K. Bean , M. Paskett , J. Kim , Z. S. Zohny , S. M. Lee , R. Zhang , K. Luo , B. Ji , A. Banks , H. M. Lee , Y. Huang , W. Z. Ray , J. A. Rogers , Nat. Med. 2018, 24, 1830. 30297910 10.1038/s41591-018-0196-2

[233] D. W. Park , J. P. Ness , S. K. Brodnick , C. Esquibel , J. Novello , F. Atry , D. H. Baek , H. Kim , J. Bong , K. I. Swanson , A. J. Suminski , K. J. Otto , R. Pashaie , J. C. Williams , Z. Ma , ACS Nano 2018, 12, 148. 29253337 10.1021/acsnano.7b04321

[234] Y. U. Cho , J. Y. Lee , U. J. Jeong , S. H. Park , S. L. Lim , K. Y. Kim , J. W. Jang , J. H. Park , H. W. Kim , H. Shin , H. Jeon , Y. M. Jung , I. J. Cho , K. J. Yu , Adv. Funct. Mater. 2021, 32.

[235] A. Burton , S. M. Won , A. K. Sohrabi , T. Stuart , A. Amirhossein , J. U. Kim , Y. Park , A. Gabros , J. A. Rogers , F. Vitale , A. G. Richardson , P. Gutruf , Microsyst. Nanoeng. 2021, 7, 62. 34567774 10.1038/s41378-021-00294-7 PMC8433476

[236] J. Park , Y. Lee , T. Y. Kim , S. Hwang , J. Seo , ACS Appl. Electron. Mater. 2022, 4, 1449.

[237] A. J. T. Teo , A. Mishra , I. Park , Y. J. Kim , W. T. Park , Y. J. Yoon , ACS Biomater. Sci. Eng. 2016, 2, 454. 33465850 10.1021/acsbiomaterials.5b00429

[238] T. A. Pham , T. K. Nguyen , R. K. Vadivelu , T. Dinh , A. Qamar , S. Yadav , Y. Yamauchi , J. A. Rogers , N. T. Nguyen , H. P. Phan , Adv. Funct. Mater. 2020, 30, 2004655.

[239] S.‐K. Kang , S.‐W. Hwang , H. Cheng , S. Yu , B. H. Kim , J.‐H. Kim , Y. Huang , J. A. Rogers , Adv. Funct. Mater. 2014, 24, 4427.

[240] J. Li , R. Li , H. Du , Y. Zhong , Y. Chen , K. Nan , S. M. Won , J. Zhang , Y. Huang , J. A. Rogers , ACS Nano 2019, 13, 660. 30608642 10.1021/acsnano.8b07806

[241] H. P. Phan , Y. Zhong , T. K. Nguyen , Y. Park , T. Dinh , E. Song , R. K. Vadivelu , M. K. Masud , J. Li , M. J. A. Shiddiky , D. Dao , Y. Yamauchi , J. A. Rogers , N. T. Nguyen , ACS Nano 2019, 13, 11572. 31433939 10.1021/acsnano.9b05168

[242] D. H. Kim , J. Viventi , J. J. Amsden , J. Xiao , L. Vigeland , Y. S. Kim , J. A. Blanco , B. Panilaitis , E. S. Frechette , D. Contreras , D. L. Kaplan , F. G. Omenetto , Y. Huang , K. C. Hwang , M. R. Zakin , B. Litt , J. A. Rogers , Nat. Mater. 2010, 9, 511. 20400953 10.1038/nmat2745 PMC3034223

[243] M. A. Escabi , H. L. Read , J. Viventi , D. H. Kim , N. C. Higgins , D. A. Storace , A. S. Liu , A. M. Gifford , J. F. Burke , M. Campisi , Y. S. Kim , A. E. Avrin , V. Spiegel Jan , Y. Huang , M. Li , J. Wu , J. A. Rogers , B. Litt , Y. E. Cohen , J. Neurophysiol. 2014, 112, 1566. 24920021 10.1152/jn.00179.2013 PMC4137255

[244] J. Li , R. Li , C.‐H. Chiang , Y. Zhong , H. Shen , E. Song , M. Hill , S. M. Won , K. J. Yu , J. M. Baek , Y. Lee , J. Viventi , Y. Huang , J. A. Rogers , Adv. Mater. Technol. 2020, 5, 1900800.

[245] G. Shin , A. M. Gomez , R. Al‐Hasani , Y. R. Jeong , J. Kim , Z. Xie , A. Banks , S. M. Lee , S. Y. Han , C. J. Yoo , J. L. Lee , S. H. Lee , J. Kurniawan , J. Tureb , Z. Guo , J. Yoon , S. I. Park , S. Y. Bang , Y. Nam , M. C. Walicki , V. K. Samineni , A. D. Mickle , K. Lee , S. Y. Heo , J. G. McCall , T. Pan , L. Wang , X. Feng , T. I. Kim , J. K. Kim , et al., Neuron 2017, 93, 509. 28132830 10.1016/j.neuron.2016.12.031 PMC5377903

[246] Y. Qiang , P. Artoni , K. J. Seo , S. Culaclii , V. Hogan , X. Zhao , Y. Zhong , X. Han , P.‐M. Wang , Y.‐K. Lo , Y. Li , H. A. Patel , Y. Huang , A. Sambangi , J. S. V. Chu , W. Liu , M. Fagiolini , H. Fang , Sci. Adv. 2018, 4, eaat0626. 30191176 10.1126/sciadv.aat0626 PMC6124910

[247] J. Li , L. Kang , Y. Yu , Y. Long , J. J. Jeffery , W. Cai , X. Wang , Nano Energy 2018, 51, 728. 30221128 10.1016/j.nanoen.2018.07.008 PMC6135531

[248] M.‐C. Choi , Y. Kim , C.‐S. Ha , Prog. Polym. Sci. 2008, 33, 581.

[249] D. H. Lee , C. H. Kim , J. Youn , J. Jeong , Biomed. Eng. Lett. 2021, 11, 97. 34150346 10.1007/s13534-021-00188-7 PMC8155150

[250] R. Caldwell , M. G. Street , R. Sharma , P. Takmakov , B. Baker , L. Rieth , Biomaterials 2020, 232, 119731. 31918225 10.1016/j.biomaterials.2019.119731

[251] R. W. Johnson , A. Hultqvist , S. F. Bent , Mater. Today 2014, 17, 236.

[252] C. Lamont , T. Grego , K. Nanbakhsh , A. Shah Idil , V. Giagka , A. Vanhoestenberghe , S. Cogan , N. Donaldson , J. Neural Eng. 2021, 18, 5003. 10.1088/1741-2552/abf0d6 PMC8208634 33752188

[253] C.‐Y. Wu , R.‐M. Liao , L.‐W. Lai , M.‐S. Jeng , D.‐S. Liu , Surf. Coat. Technol. 2012, 206, 4685.

[254] S. H. Ahn , J. Jeong , S. J. Kim , Micromachines (Basel) 2019, 10. 10.3390/mi10080508 PMC6723304 31370259

[255] K. Kim , M. Van Gompel , K. Wu , G. Schiavone , J. Carron , F. Bourgeois , S. P. Lacour , Y. Leterrier , Small 2021, 17, 2103039. 10.1002/smll.202103039 34477315

[256] J. Jeong , F. Laiwalla , J. Lee , R. Ritasalo , M. Pudas , L. Larson , V. Leung , A. Nurmikko , Adv. Funct. Mater. 2018, 29, 1806440.

[257] E. Song , Y. K. Lee , R. Li , J. Li , X. Jin , K. J. Yu , Z. Xie , H. Fang , Y. Zhong , H. Du , J. Zhang , G. Fang , Y. Kim , Y. Yoon , M. A. Alam , Y. Mei , Y. Huang , J. A. Rogers , Adv. Funct. Mater. 2017, 28, 1702284.

[258] S. Minnikanti , G. Diao , J. J. Pancrazio , X. Xie , L. Rieth , F. Solzbacher , N. Peixoto , Acta Biomater. 2014, 10, 960. 24185000 10.1016/j.actbio.2013.10.031

[259] Q. Zheng , Y. Zou , Y. Zhang , Z. Liu , B. Shi , X. Wang , Y. Jin , H. Ouyang , Z. Li , Z. L. Wang , Sci. Adv. 2016, 2, e1501478. 26973876 10.1126/sciadv.1501478 PMC4783121

[260] C. Li , S. Yang , B. Mader , Coatings 2019, 9, 579.

[261] H. Fang , J. Zhao , K. J. Yu , E. Song , A. B. Farimani , C. H. Chiang , X. Jin , Y. Xue , D. Xu , W. Du , K. J. Seo , Y. Zhong , Z. Yang , S. M. Won , G. Fang , S. W. Choi , S. Chaudhuri , Y. Huang , M. A. Alam , J. Viventi , N. R. Aluru , J. A. Rogers , Proc. Natl. Acad. Sci. USA 2016, 113, 11682. 27791052 10.1073/pnas.1605269113 PMC5081656

[262] Y. K. Lee , K. J. Yu , Y. Kim , Y. Yoon , Z. Xie , E. Song , H. Luan , X. Feng , Y. Huang , J. A. Rogers , ACS Appl. Mater. Interfaces 2017, 9, 42633. 29178781 10.1021/acsami.7b15302 PMC6800003

[263] E. Song , H. Fang , X. Jin , J. Zhao , C. Jiang , K. J. Yu , Y. Zhong , D. Xu , J. Li , G. Fang , H. Du , J. Zhang , J. M. Park , Y. Huang , M. A. Alam , Y. Mei , J. A. Rogers , Adv. Electron. Mater. 2017, 3, 1700077.

[264] H. Fang , K. J. Yu , C. Gloschat , Z. Yang , C. H. Chiang , J. Zhao , S. M. Won , S. Xu , M. Trumpis , Y. Zhong , E. Song , S. W. Han , Y. Xue , D. Xu , G. Cauwenberghs , M. Kay , Y. Huang , J. Viventi , I. R. Efimov , J. A. Rogers , Nat. Biomed. Eng. 2017, https://do.org/10.1038/s41551-017-0038. 10.1038/s41551-017-0038 PMC5552067 28804678

[265] J. Viventi , D. H. Kim , L. Vigeland , E. S. Frechette , J. A. Blanco , Y. S. Kim , A. E. Avrin , V. R. Tiruvadi , S. W. Hwang , A. C. Vanleer , D. F. Wulsin , K. Davis , C. E. Gelber , L. Palmer , J. Van der Spiegel , J. Wu , J. Xiao , Y. Huang , D. Contreras , J. A. Rogers , B. Litt , Nat. Neurosci. 2011, 14, 1599. 22081157 10.1038/nn.2973 PMC3235709

[266] Y. K. Lee , K. J. Yu , E. Song , A. Barati Farimani , F. Vitale , Z. Xie , Y. Yoon , Y. Kim , A. Richardson , H. Luan , Y. Wu , X. Xie , T. H. Lucas , K. Crawford , Y. Mei , X. Feng , Y. Huang , B. Litt , N. R. Aluru , L. Yin , J. A. Rogers , ACS Nano 2017, 11, 12562. 29178798 10.1021/acsnano.7b06697 PMC5830089

[267] H. Zhuang , N. Yang , L. Zhang , R. Fuchs , X. Jiang , ACS Appl. Mater. Interfaces 2015, 7, 10886. 25939808 10.1021/acsami.5b02024

[268] P. Xing , D. Ma , K. J. A. Ooi , J. W. Choi , A. M. Agarwal , D. Tan , A. C. S. Photonics , 2019, 6, 1162.

[269] S. F. Cogan , D. J. Edell , A. A. Guzelian , Y. P. Liu , R. Edell , J. Biomed. Mater. Res., Part A 2003, 67A, 856. 10.1002/jbm.a.10152 14613234

[270] L. Jiang , Y. Yang , R. Chen , G. Lu , R. Li , D. Li , M. S. Humayun , K. K. Shung , J. Zhu , Y. Chen , Q. Zhou , Nano Energy 2019, 56, 216. 31475091 10.1016/j.nanoen.2018.11.052 PMC6717511

[271] L. Xu , S. R. Gutbrod , Y. Ma , A. Petrossians , Y. Liu , R. C. Webb , J. A. Fan , Z. Yang , R. Xu , J. J. Whalen, 3rd , J. D. Weiland , Y. Huang , I. R. Efimov , J. A. Rogers , Adv. Mater. 2015, 27, 1731. 25641076 10.1002/adma.201405017 PMC4527319

[272] D. Khodagholy , J. N. Gelinas , T. Thesen , W. Doyle , O. Devinsky , G. G. Malliaras , G. Buzsaki , Nat. Neurosci. 2015, 18, 310. 25531570 10.1038/nn.3905 PMC4308485

[273] C. Y. Kim , M. J. Ku , R. Qazi , H. J. Nam , J. W. Park , K. S. Nam , S. Oh , I. Kang , J. H. Jang , W. Y. Kim , J. H. Kim , J. W. Jeong , Nat. Commun. 2021, 12, 535. 33483493 10.1038/s41467-020-20803-y PMC7822865

[274] S. Daghighi , J. Sjollema , H. C. van der Mei , H. J. Busscher , E. T. Rochford , Biomaterials 2013, 34, 8013. 23915949 10.1016/j.biomaterials.2013.07.044

[275] C. Li , C. Guo , V. Fitzpatrick , A. Ibrahim , M. J. Zwierstra , P. Hanna , A. Lechtig , A. Nazarian , S. J. Lin , D. L. Kaplan , Nat. Rev. Mater. 2019, 5, 61.

[276] A. A. La Mattina , S. Mariani , G. Barillaro , Adv. Sci. 2020, 7, 1902872. 10.1002/advs.201902872 PMC7029671 32099766

[277] E. J. Dierings de Souza , D. H. Kringel , A. R. Guerra Dias , E. da Rosa Zavareze , Carbohydr. Polym. 2021, 265, 118068. 33966832 10.1016/j.carbpol.2021.118068

[278] L. N. Woodard , M. A. Grunlan , ACS Macro Lett. 2018, 7, 976. 30705783 10.1021/acsmacrolett.8b00424 PMC6350899

[279] G. Lee , S.‐K. Kang , S. M. Won , P. Gutruf , Y. R. Jeong , J. Koo , S.‐S. Lee , J. A. Rogers , J. S. Ha , Adv. Energy Mater. 2017, 7, 1700157.

[280] Y. S. Choi , Y. Y. Hsueh , J. Koo , Q. Yang , R. Avila , B. Hu , Z. Xie , G. Lee , Z. Ning , C. Liu , Y. Xu , Y. J. Lee , W. Zhao , J. Fang , Y. Deng , S. M. Lee , A. Vazquez‐Guardado , I. Stepien , Y. Yan , J. W. Song , C. Haney , Y. S. Oh , W. Liu , H. J. Yoon , A. Banks , M. R. MacEwan , G. A. Ameer , W. Z. Ray , Y. Huang , T. Xie , et al., Nat. Commun. 2020, 11, 5990. 33239608 10.1038/s41467-020-19660-6 PMC7688647

[281] Y. Liu , X. Du , H. Wang , Y. Yuan , L. Wei , X. Liu , A. Sun , Y. Li , Front. Mater. Sci. 2022, 16, 220598.

[282] P. Nellepalli , T. Patel , J. K. Oh , Macromol. Rapid Commun. 2021, 42, 2100391. 10.1002/marc.202100391 34418209

[283] C. Wang , T. Yokota , T. Someya , Chem. Rev. 2021, 121, 2109. 33460327 10.1021/acs.chemrev.0c00897

[284] C. Peniche , W. Argüelles‐Monal , H. Peniche , N. Acosta , Macromol. Biosci. 2003, 3, 511.

[285] A. B. Balaji , H. Pakalapati , M. Khalid , R. Walvekar , H. Siddiqui , in Biodegradable and Biocompatible Polymer Composites, Woodhead Publishing, Sawston, Cambridge 2018, p. 3.

[286] A. C. Patil , Z. Xiong , N. V. Thakor , Small Methods 2020, 4.

[287] S.‐W. Hwang , H. Tao , D.‐H. Kim , H. Cheng , J.‐K. Song , E. Rill , M. A. Brenckle , B. Panilaitis , S. M. Won , Y.‐S. Kim , Y. M. Song , K. J. Yu , A. Ameen , R. Li , Y. Su , M. Yang , D. L. Kaplan , M. R. Zakin , M. J. Slepian , Y. Huang , F. G. Omenetto , J. A. Rogers , Science 2012, 337, 1640. 23019646 10.1126/science.1226325 PMC3786576

[288] X. Tong , W. Pan , T. Su , M. Zhang , W. Dong , X. Qi , React. Funct. Polym. 2020, 148, 104501.

[289] E. S. Place , J. H. George , C. K. Williams , M. M. Stevens , Chem. Soc. Rev. 2009, 38, 1139. 19421585 10.1039/b811392k

[290] K. J. Yu , D. Kuzum , S. W. Hwang , B. H. Kim , H. Juul , N. H. Kim , S. M. Won , K. Chiang , M. Trumpis , A. G. Richardson , H. Cheng , H. Fang , M. Thomson , H. Bink , D. Talos , K. J. Seo , H. N. Lee , S. K. Kang , J. H. Kim , J. Y. Lee , Y. Huang , F. E. Jensen , M. A. Dichter , T. H. Lucas , J. Viventi , B. Litt , J. A. Rogers , Nat. Mater. 2016, 15, 782. 27088236 10.1038/nmat4624 PMC4919903

[291] J. Shin , Y. Yan , W. Bai , Y. Xue , P. Gamble , L. Tian , I. Kandela , C. R. Haney , W. Spees , Y. Lee , M. Choi , J. Ko , H. Ryu , J. K. Chang , M. Pezhouh , S. K. Kang , S. M. Won , K. J. Yu , J. Zhao , Y. K. Lee , M. R. MacEwan , S. K. Song , Y. Huang , W. Z. Ray , J. A. Rogers , Nat. Biomed. Eng. 2019, 3, 37. 30932064 10.1038/s41551-018-0300-4

[292] J. H. Koo , J.‐K. Song , D.‐H. Kim , D. Son , ACS Mater. Lett. 2021, 3, 1528.

[293] S. P. Lacour , G. Courtine , J. Guck , Nat. Rev. Mater. 2016, 1, 16063.

[294] Y. Liu , Y. H. Hsu , A. P. Huang , S. H. Hsu , ACS Appl. Mater. Interfaces 2020, 12, 40108. 32808527 10.1021/acsami.0c11433

[295] A. Carnicer‐Lombarte , S. T. Chen , G. G. Malliaras , D. G. Barone , Front. Bioeng. Biotechnol. 2021, 9, 622524. 33937212 10.3389/fbioe.2021.622524 PMC8081831

[296] H. Seo , S. I. Han , K. I. Song , D. Seong , K. Lee , S. H. Kim , T. Park , J. H. Koo , M. Shin , H. W. Baac , O. K. Park , S. J. Oh , H. S. Han , H. Jeon , Y. C. Kim , D. H. Kim , T. Hyeon , D. Son , Adv. Mater. 2021, 33, 2007346. 10.1002/adma.202007346 33739558

[297] D. H. Kim , N. Lu , R. Ghaffari , Y. S. Kim , S. P. Lee , L. Xu , J. Wu , R. H. Kim , J. Song , Z. Liu , J. Viventi , B. de Graff , B. Elolampi , M. Mansour , M. J. Slepian , S. Hwang , J. D. Moss , S. M. Won , Y. Huang , B. Litt , J. A. Rogers , Nat. Mater. 2011, 10, 316. 21378969 10.1038/nmat2971 PMC3132573

[298] J. Liu , X. Zhang , Y. Liu , M. Rodrigo , P. D. Loftus , J. Aparicio‐Valenzuela , J. Zheng , T. Pong , K. J. Cyr , M. Babakhanian , J. Hasi , J. Li , Y. Jiang , C. J. Kenney , P. J. Wang , A. M. Lee , Z. Bao , Proc. Natl. Acad. Sci. USA 2020, 117, 14769. 32541030 10.1073/pnas.2000207117 PMC7334471

[299] S. I. Park , G. Shin , J. G. McCall , R. Al‐Hasani , A. Norris , L. Xia , D. S. Brenner , K. N. Noh , S. Y. Bang , D. L. Bhatti , K. I. Jang , S. K. Kang , A. D. Mickle , G. Dussor , T. J. Price , R. W. t. Gereau , M. R. Bruchas , J. A. Rogers , Proc. Natl. Acad. Sci. USA 2016, 113, E8169. 27911798 10.1073/pnas.1611769113 PMC5167187

[300] D. S. Kim , Y. W. Choi , A. Shanmugasundaram , Y. J. Jeong , J. Park , N. E. Oyunbaatar , E. S. Kim , M. Choi , D. W. Lee , Nat. Commun. 2020, 11, 535. 31988308 10.1038/s41467-019-14019-y PMC6985253

[301] S. Liang , Y. Zhang , H. Wang , Z. Xu , J. Chen , R. Bao , B. Tan , Y. Cui , G. Fan , W. Wang , W. Wang , W. Liu , Adv. Mater. 2018, 30, 1704235. 10.1002/adma.201704235 29687502

[302] Y. Liu , H. Zhou , W. Zhou , S. Meng , C. Qi , Z. Liu , T. Kong , Adv. Energy Mater. 2021, 11, 2101329.

[303] M. Mehrasa , M. A. Asadollahi , K. Ghaedi , H. Salehi , A. Arpanaei , Int. J. Biol. Macromol. 2015, 79, 687. 26045092 10.1016/j.ijbiomac.2015.05.050

[304] E. Vey , C. Rodger , J. Booth , M. Claybourn , A. F. Miller , A. Saiani , Polym. Degrad. Stab. 2011, 96, 1882.

[305] Y. S. Choi , R. T. Yin , A. Pfenniger , J. Koo , R. Avila , K. Benjamin Lee , S. W. Chen , G. Lee , G. Li , Y. Qiao , A. Murillo‐Berlioz , A. Kiss , S. Han , S. M. Lee , C. Li , Z. Xie , Y. Y. Chen , A. Burrell , B. Geist , H. Jeong , J. Kim , H. J. Yoon , A. Banks , S. K. Kang , Z. J. Zhang , C. R. Haney , A. V. Sahakian , D. Johnson , T. Efimova , Y. Huang , et al., Nat. Biotechnol. 2021, 39, 1228. 34183859 10.1038/s41587-021-00948-x PMC9270064

[306] Z. Wang , L. Yu , M. Ding , H. Tan , J. Li , Q. Fu , Polym. Chem. 2011, 2, 601.

[307] S. H. Hsu , L. G. Dai , Y. M. Hung , N. T. Dai , Int. J. Nanomed. 2018, 13, 5485. 10.2147/IJN.S169825 PMC6149831 30271142

[308] K. Tapily , J. E. Jakes , D. S. Stone , P. Shrestha , D. Gu , H. Baumgart , A. A. Elmustafa , J. Electrochem. Soc. 2008, 155, H545.

[309] C. Kathe , F. Michoud , P. Schonle , A. Rowald , N. Brun , J. Ravier , I. Furfaro , V. Paggi , K. Kim , S. Soloukey , L. Asboth , T. H. Hutson , I. Jelescu , A. Philippides , N. Alwahab , J. Gandar , D. Huber , C. I. De Zeeuw , Q. Barraud , Q. Huang , S. P. Lacour , G. Courtine , Nat. Biotechnol. 2022, 40, 198. 34580478 10.1038/s41587-021-01019-x PMC7612390

[310] B. Wang , W. Huang , L. Chi , M. Al‐Hashimi , T. J. Marks , A. Facchetti , Chem. Rev. 2018, 118, 5690. 29785854 10.1021/acs.chemrev.8b00045

[311] M. Xie , G. Yao , T. Zhang , Q. Wang , X. Mo , Q. Dong , W. Lou , F. Lu , T. Pan , M. Gao , D. Jiang , K. Zhao , Y. Lin , J. Nanobiotechnol. 2022, 20, 202. 10.1186/s12951-022-01415-8 PMC9044588 35477463

[312] Q. Lu , X. Hu , X. Wang , J. A. Kluge , S. Lu , P. Cebe , D. L. Kaplan , Acta Biomater. 2010, 6, 1380. 19874919 10.1016/j.actbio.2009.10.041 PMC2830340

[313] K. Tsuchiya , H. Masunaga , K. Numata , Biomacromolecules 2017, 18, 1002. 28117990 10.1021/acs.biomac.6b01891

[314] Q. Lu , B. Zhang , M. Li , B. Zuo , D. L. Kaplan , Y. Huang , H. Zhu , Biomacromolecules 2011, 12, 1080. 21361368 10.1021/bm101422j PMC3404841

[315] T.‐H. Kim , W. M. Choi , D.‐H. Kim , M. A. Meitl , E. Menard , H. Jiang , J. A. Carlisle , J. A. Rogers , Adv. Mater. 2008, 20, 2171.

[316] H. Tada , A. E. Kumpel , R. E. Lathrop , J. B. Slanina , P. Nieva , P. Zavracky , I. N. Miaoulis , P. Y. Wong , J. Appl. Phys. 2000, 87, 4189.

[317] W. Fang , J. Micromech. Microeng. 1999, 9, 230.

[318] S.‐W. Hwang , D.‐H. Kim , H. Tao , T.‐i. Kim , S. Kim , K. J. Yu , B. Panilaitis , J.‐W. Jeong , J.‐K. Song , F. G. Omenetto , J. A. Rogers , Adv. Funct. Mater. 2013, 23, 4087.

[319] A. Khan , J. Philip , P. Hess , J. Appl. Phys. 2004, 95, 1667.

[320] P. Walsh , A. Omeltchenko , R. K. Kalia , A. Nakano , P. Vashishta , S. Saini , Appl. Phys. Lett. 2003, 82, 118.

[321] A. Zerr , M. Kempf , M. Schwarz , E. Kroke , M. Göken , R. Riedel , J. Am. Ceram. Soc. 2002, 85, 86.

[322] E. Song , H. Fang , X. Jin , J. Zhao , C. Jiang , K. J. Yu , Y. Zhong , D. Xu , J. Li , G. Fang , H. Du , J. Zhang , J. M. Park , Y. Huang , M. A. Alam , Y. Mei , J. A. Rogers , Adv. Electron. Mater. 2017, 3, 1700077.

[323] A. K. Katiyar , A. A. Davidson , H. Jang , Y. Hwangbo , B. Han , S. Lee , Y. Hagiwara , T. Shimada , H. Hirakata , T. Kitamura , J. H. Ahn , Nanoscale 2019, 11, 15184. 31380876 10.1039/c9nr03995c

[324] X. Zhu , J. Lu , H. Pan , Z. Zheng , X. Pi , Y. Zhao , Adv. Mater. Interfaces 2017, 4, 1770529.

[325] M. K. Niranjan , Mater. Res. Express 2015, 2, 6302.

[326] M. Berdova , X. Liu , C. Wiemer , A. Lamperti , G. Tallarida , E. Cianci , M. Fanciulli , S. Franssila , J. Vac. Sci. Technol., A 2016, 34, 1510.

[327] K. Tapily , J. Jakes , P. R. Shrestha , D. Gu , H. Baumgart , A. Elmustafa , ECS Trans. 2008, 16, 269.

[328] J.‐W. Han , H.‐J. Kang , J.‐Y. Kim , G.‐Y. Kim , D.‐S. Seo , Jpn. J. Appl. Phys. 2006, 45, 9203.

[329] S. Packer , N. Mercado , A. Haridat , Cold Spring Harb. Perspect. Med. 2019, 9, 4363. 10.1101/cshperspect.a034363 PMC6771365 30478096

[330] L. Drew , Nature 2019, 571, S19. 31341310 10.1038/d41586-019-02214-2

